# Marine Brominated Tyrosine Alkaloids as Promising Inhibitors of SARS-CoV-2

**DOI:** 10.3390/molecules26206171

**Published:** 2021-10-13

**Authors:** Amr El-Demerdash, Afnan Hassan, Tarek Mohamed Abd El-Aziz, James D. Stockand, Reem K. Arafa

**Affiliations:** 1 Department of Metabolic Biology & Biological Chemistry, The John Innes Center, Norwich Research Park, Norwich NR4 7UH, UK; 2 Division of Organic Chemistry, Department of Chemistry, Faculty of Science, Mansoura University, Mansoura 35516, Egypt; 3 Drug Design and Discovery Laboratory, Helmy Institute for Medical Sciences, Zewail City of Science and Technology, Giza 12578, Egypt; afhassan@zewailcity.edu.eg; 4 Biomedical Sciences Program, University of Science and Technology, Zewail City of Science and Technology, Giza 12578, Egypt; 5 Zoology Department, Faculty of Science, Minia University, El-Minia 61519, Egypt; mohamedt1@uthscsa.edu; 6 Department of Cellular and Integrative Physiology, University of Texas Health Science Center at San Antonio, San Antonio, TX 78229, USA

**Keywords:** SARS-CoV-2, virtual screening, molecular docking, molecular dynamics simulation, ADME/Tox, brominated tyrosine alkaloids

## Abstract

There have been more than 150 million confirmed cases of SARS-CoV-2 since the beginning of the pandemic in 2019. By June 2021, the mortality from such infections approached 3.9 million people. Despite the availability of a number of vaccines which provide protection against this virus, the evolution of new viral variants, inconsistent availability of the vaccine around the world, and vaccine hesitancy, in some countries, makes it unreasonable to rely on mass vaccination alone to combat this pandemic. Consequently, much effort is directed to identifying potential antiviral treatments. Marine brominated tyrosine alkaloids are recognized to have antiviral potential. We test here the antiviral capacity of fourteen marine brominated tyrosine alkaloids against five different target proteins from SARS-CoV-2, including main protease (M^pro^) (PDB ID: 6lu7), spike glycoprotein (PDB ID: 6VYB), nucleocapsid phosphoprotein (PDB ID: 6VYO), membrane glycoprotein (PDB ID: 6M17), and non-structural protein 10 (nsp10) (PDB ID: 6W4H). These marine alkaloids, particularly the hexabrominated compound, fistularin-3, shows promising docking interactions with predicted binding affinities (S-score = −7.78, −7.65, −6.39, −6.28, −8.84 Kcal/mol) for the main protease (M^pro^) (PDB ID: 6lu7), spike glycoprotein (PDB ID: 6VYB), nucleocapsid phosphoprotein (PDB ID: 6VYO), membrane glycoprotein (PDB ID: 6M17), and non-structural protein 10 (nsp10) (PDB ID: 6W4H), respectively, where it forms better interactions with the protein pockets than the native interaction. It also shows promising molecular dynamics, pharmacokinetics, and toxicity profiles. As such, further exploration of the antiviral properties of fistularin-3 against SARS-CoV-2 is merited.

## 1. Introduction

The 2019 novel coronavirus disease (COVID-19), caused by severe acute respiratory syndrome coronavirus 2 (SARS-CoV-2), has significantly impacted global health and economics [1]. The signs and symptoms of COVID-19 are grouped into three categories according to the severity of the infection and mortality: mild, severe, and critical. The majority of COVID-19 patients, 80%, experience mild symptoms and recover. Severe symptoms appear in 13.8% of cases and 6.1% become critically ill [2,3].

SARS-CoV-2 is the seventh coronavirus known to infect humans, but the only one, to date, that has caused a pandemic [4,5]. SARS-CoV-2 was first detected in 2019 in Wuhan, China, and possibly originated from a recombination event in an ancestor of SARS-CoV-2, a horseshoe bat coronavirus, around 11 years ago via zoonotic transmission from the pangolins [6,7].

**Figure 1:**
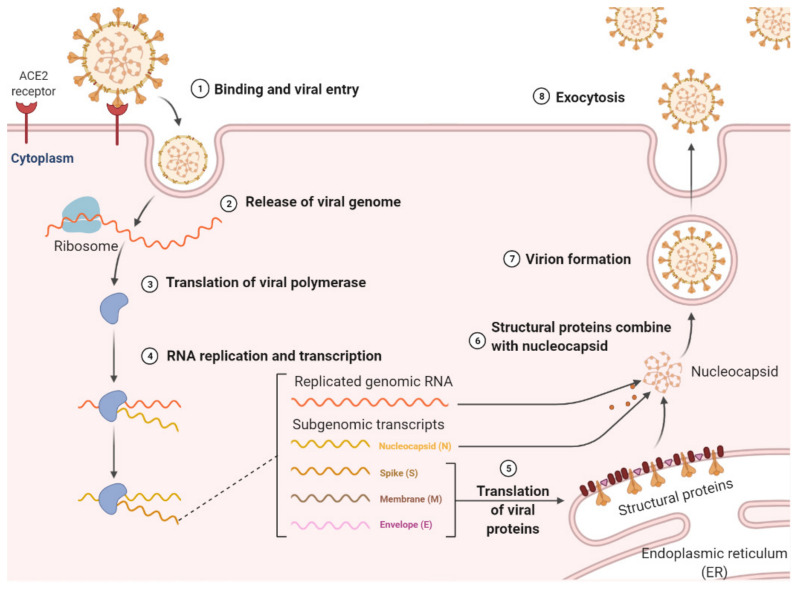
Life cycle of SARS-CoV-2. SARS-CoV-2 attaches to the host cell through the ACE2 receptor. It is internalized and its RNA is released into the cytoplasm where genome replication and translation of viral structural and accessory proteins occurs. After assembly, mature virion particles are released by exocytosis. Created with BioRender.com (accessed on 28 June 2021).

Host cell entry is the first step in the viral life cycle. The first step in the life cycle of SARS-CoV-2 is the attachment of the viral particle via the receptor-binding domain (RBD) of its S protein (see below) to the angiotensin-converting enzyme-2 (ACE-2) receptor on the plasma membrane of the pulmonary alveolar epithelial cells and capillary endothelial cells. In some instances, this ultimately leads to severe acute respiratory failure (Figure 1) [8,9].

Similar to other coronaviruses (CoVs), the size of the SARS-CoV-2 genome is approximately 30 Kb and encodes four main structural proteins, including the Spike glycoprotein (S protein), Envelope (E) protein, Membrane (M) protein, and Nucleocapsid (N protein) [10]. In addition to these four main structural proteins, the SARS-CoV-2 genome codes for sixteen non-structural proteins (NSP 1−16) [11]. Together these proteins facilitate the replication of the virus in the host cell. While effective vaccines and vaccination programs are ongoing, new viral variants are emerging, and infections, hospitalizations and deaths from COVID-19 continue. Consequently, it is of paramount importance to develop effective inhibitors of SARS-CoV-2.

Natural products are commonly recognized for their therapeutic potentials [12,13,14]. Since the discovery of the first marine nucleosides, spongothymidine and spongouridine, from the Caribbean marine sponge, *Cryptotethya crypta*, in the early 1950s, a new era of the exploitation of bioactive marine natural products (MNPs) has emerged [15,16,17]. In the 1970s, synthetic organic chemistry efforts created the initial marine-based drugs cytarabine, Cytosar-U^®^, and Depocyst^®^, approved by the FDA for cancer treatment; and vidarabine, and Vira-A^®^, approved as antiviral agents [18]. Marine natural products continue to serve as robust and sustainable pipelines for drug leads. In particular, marine sponges are known to produce numerous MNPs which are possibly suitable for use as drugs [19,20,21,22,23,24]. Brominated tyrosine alkaloids (BTAs) are a distinct class of sponge-derived secondary metabolites which are biosynthetically derived from tyrosine and feature structural diversities with myriad biomedical applications. The majority of this class of MNPs are isolated from marine sponges belonging to the order Verongiida [25]. BTAs demonstrated diverse bioactivities including cytotoxicity [26], antifungal [27], antibacterial [28], and acetylcholinesterase (AChE) inhibition [29]. Additionally, a considerable number of BTAs have antiviral activity. For instance, moloka′iamine (**1**), a dibrominated compound from the Verongida sponge, is 90% inhibitory (at a dose of 10 μg/mL) against HSV-II [30]. Mololipids (**2**), a series of brominated tyrosine containing lipids isolated from a Verongida sponge from the south shore of O′ahu island, Hawaii, has a selective antiviral activity against HIV-1 (ED_50_ of 52.2 μM) in the absence of generalized cytotoxicity (at IC_50_ > 100 μg/mL) against human peripheral blood mononuclear cells. This highlights why these types of brominated lipids are promising antiviral agents. Fistularin-3 (**3**) and 11-ketofistularin (**4**) are hexabrominated compounds from the *Aplysina archeri* marine sponges. They have an activity against the feline leukemia virus with an ED_50_ of 22 μM (4.8 μg/200 μL) and 42 μM (9.3 μg/200 μL), respectively. Additionally, at 100 μg/200 μL, which is the highest concentration tested for cytotoxicity, neither compound is toxic. While these brominated alkaloids are less active than 3′-azido-3′-deoxythymidine (AZT, ED_50_ of 0.10 μM), they are comparable to 2′,3′-dideoxycytidine (ddCyd, ED_50_ of 15 μM) in similar assays [31]. Moreover, fistularin-3 (**3**) also exhibited an anti-HIV-1 activity with an EC_50_ of 6.9 μM [32]. Psammaplysin D (**5**) is a polybrominated compound from sponges belonging to the genus *Aplysinella* (Order: Verongida, Family: Aplysinellidae) that features a spirooxepinisoxazoline scaffold. It displays an anti-HIV activity against the Haitian RF strain of HIV-I with a 51% inhibition at 0.1 μg/mL [33].

Because of these antiviral activities and the pressing need to immediately identify the functional antivirals against COVID-19, we quantified the potential interactions of fourteen (**1–14**) structurally diverse marine brominated tyrosine alkaloids with five SARS-CoV-2 protein targets. This was accomplished through a virtual screening of their docking predicted affinities, molecular dynamics and structure–activity relations [34,35,36,37].

## 2. Results and Discussion

### 2.1. Docking Validation and High Throughput Virtual Screening of BTAs

**Figure 2:**
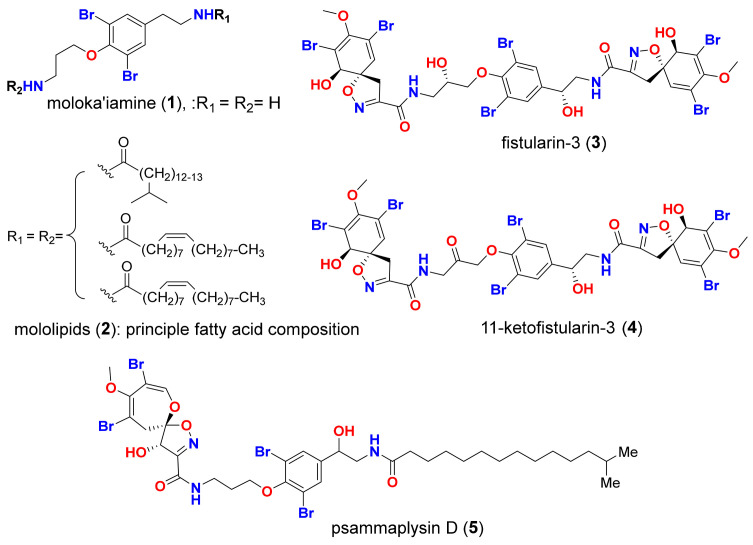
Reported antiviral bromotyrosine compounds **1–5**.

**Figure 3:**
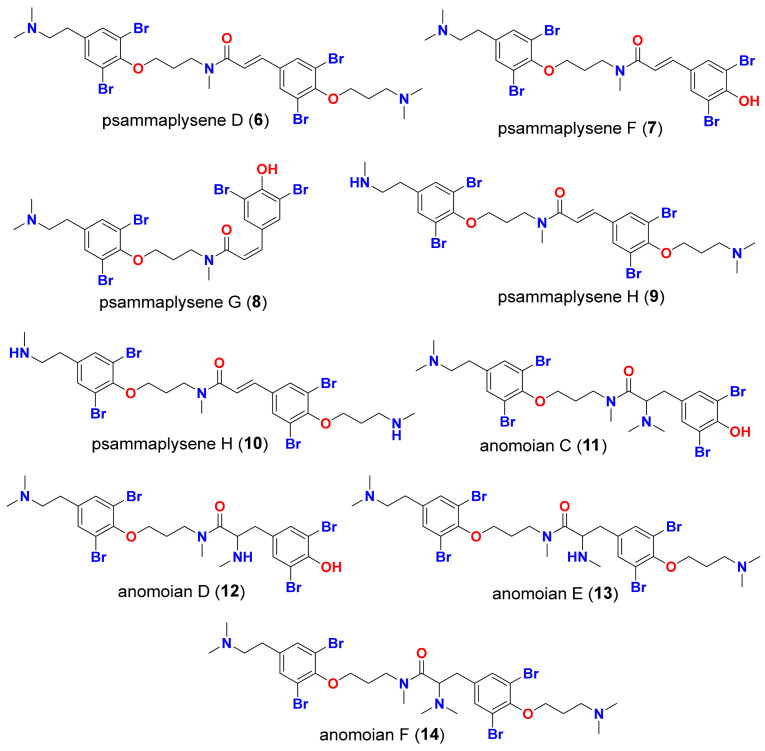
Isolated bromotyrosine derivatives (**6–14**) from the marine sponge, *Suberea ianthelliformis*.

In this work, a high throughput virtual screening of a library consisting of fourteen marine BTAs, five bromotyrosine compounds with established antiviral properties (**1**–**5**, Figure 2), and nine bromotyrosine derivatives (**6**–**14**, Figure 3) from the French Polynesian marine sponge, *Suberea ianthelliformis*, was performed against five SARS-CoV-2 target proteins. The binding potentials of the compounds in this screening library were indicated as S-scores and compared to those of the co-crystallized native ligand for each of the five target proteins for the aim of docking validation and a cross-reference comparison, as shown in Table 1.

For the SARS-CoV-2 Main protease (M^Pro^), compound **13** had a better S-score compared to the co-crystallized ligand (S-scores −8.54 vs. −8.25 Kcal/mol, respectively). The other library compounds also demonstrated effective, predicted binding affinities with S-scores between −5.97 and −8.02 Kcal/mol.

The S-scores for all fourteen library compounds were greater for the spike glycoprotein (−5.14 to −7.65 Kcal/mol) compared to the spike glycoprotein co-crystallized with a native ligand (S-score = −4.55 Kcal/mol), with compound **3** once more having the best predicted binding affinity (S-score = −7.65 Kcal/mol).

Compound **1** had a similar predicted binding affinity with nucleocapsid phosphoprotein compared to the co-crystallized native ligand (−4.33 vs. −4.44 Kcal/mol, respectively). The other thirteen compounds had higher S-scores (−5.10 to−7.04 Kcal/mol) indicating greater binding affinities, with compound **2** having the highest binding score.

For the SARS-CoV-2 membrane glycoprotein; the spike protein bound to the PD of ACE2 with a dissociation constant of ~ 15 nM and compound **3** having the greatest predicted binding affinity (S-score = −6.28 Kcal/mol).

All library compounds had similar predicted binding affinities (−7.06 to −9.77 Kcal/mol) for the non-structural protein 10 (nsp10) as the co-crystallized native ligand (−9.43 Kcal/mol) except for compound **1** which had a substantially lower S-score (−5.38 Kcal/mol).

The 2D interactions of the screening library compounds compared to those of the native co-crystallized ligands of the five SARS-CoV-2 target proteins were studied with binding data for each compound displayed in Table 2.

According to Table 2, the hexabrominated compound, fistularin-3 (**3**), and the co-crystalized native ligand showed similar interactions with the active pocket of M^Pro^. Both exhibited two H-bond donor interactions with Glu 166 and a pi-H interaction with the active pocket. Similarly, 11-ketofistularin (**4**) formed a H-bond donor interaction between O78 of the ligand and Glu 166 in the pocket and a H-bond acceptor between O40 of the ligand and Gly 143 of M^Pro^.

Among the nine bromotyrosine derivatives from the marine sponge, *Suberea ianthelliformis* (compounds **6**–**14**), compounds **10**, **13,** and **14** showed the best interactions with the active pocket of M^Pro^. Psammaplysene H (**10**) exhibited four interactions: three H-bond donors, one between N62 of the ligand and Thr 190 of the receptor, one between Br68 of the ligand and Glu 166 in the pocket, and one between Br69 of the ligand and Cys 145 in the pocket, as well as a H-pi interaction between C7 of the ligand and His 41 of the pocket. Anomoian E (**13**) also formed two interactions: a H-bond donor between Br37 of the ligand and Asn 142 of the receptor and a pi-H interaction between the ligand’s six-membered ring and Glu 166.

**Figure 4:**
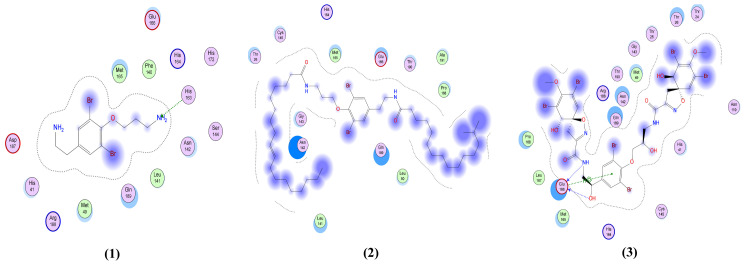

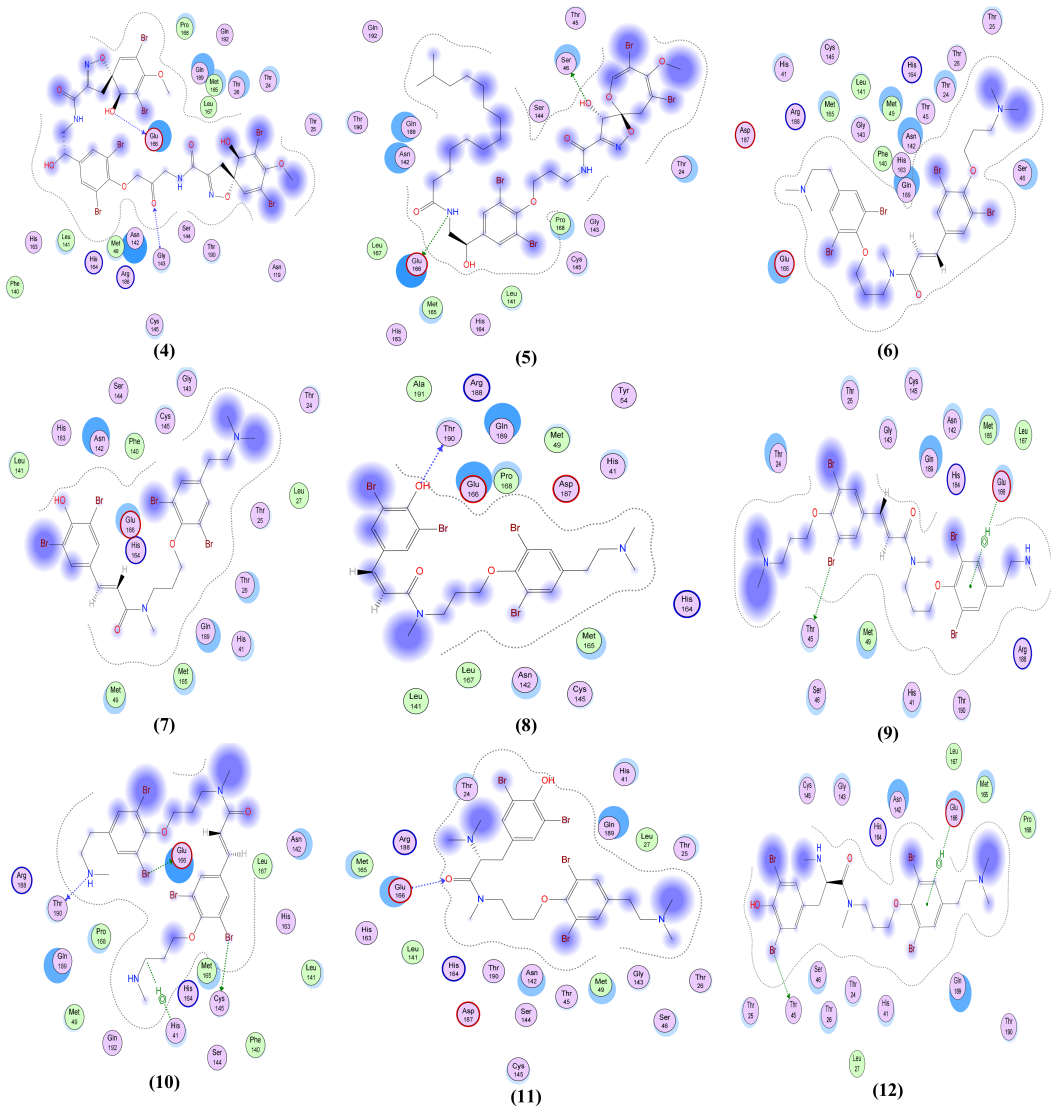

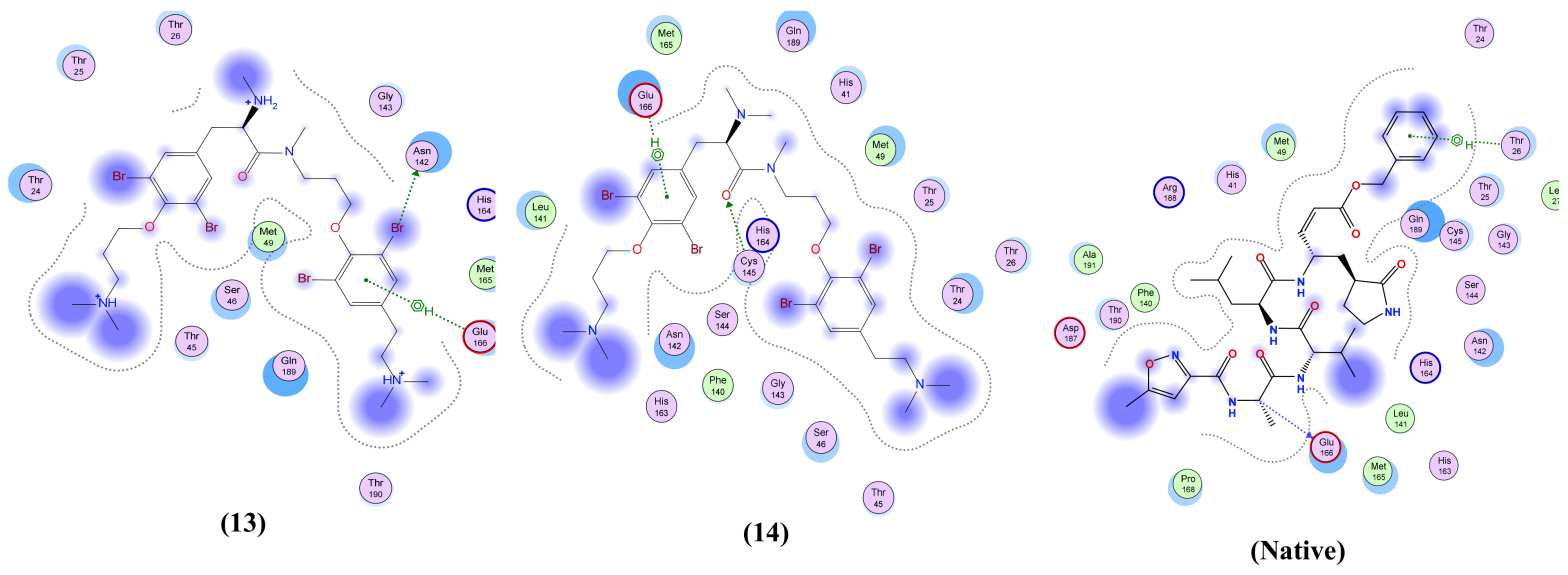
2D interactions between compounds **1–14** and the native ligand with M^Pro^ (PDB ID: 6lu7).

Similarly, anomoian F (**14**) also showed two interactions: a H-bond acceptor between O35 of the ligand and Cys 145 in the protein pocket, and a pi-H interaction between the ligand’s six-membered ring and Glu 166. The 2D interactions of the fourteen test compounds from the screening library, in addition to the native ligand with M^Pro^, are shown in Figure 4.

**Figure 5:**
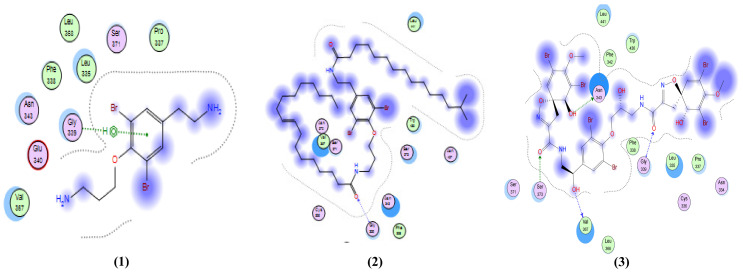

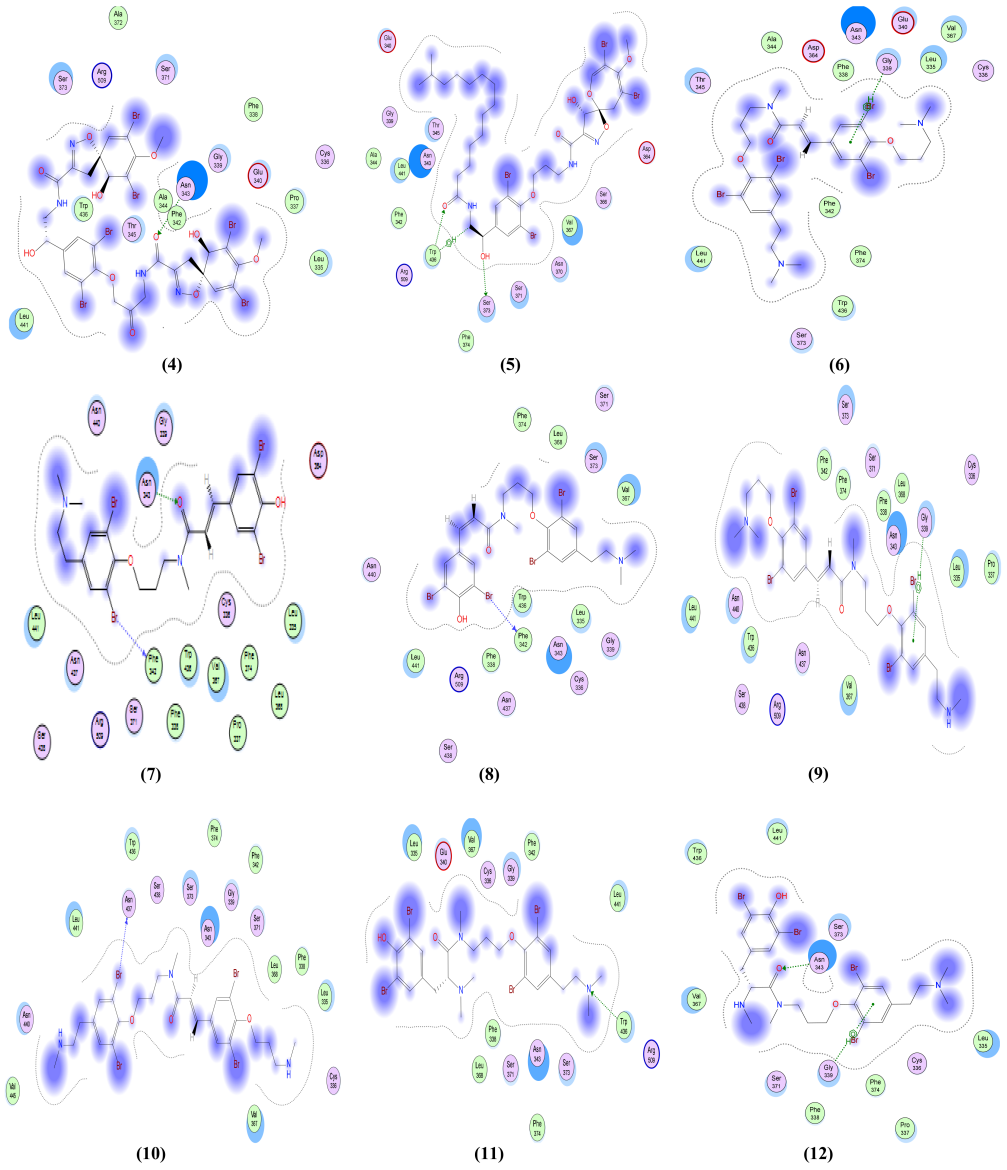

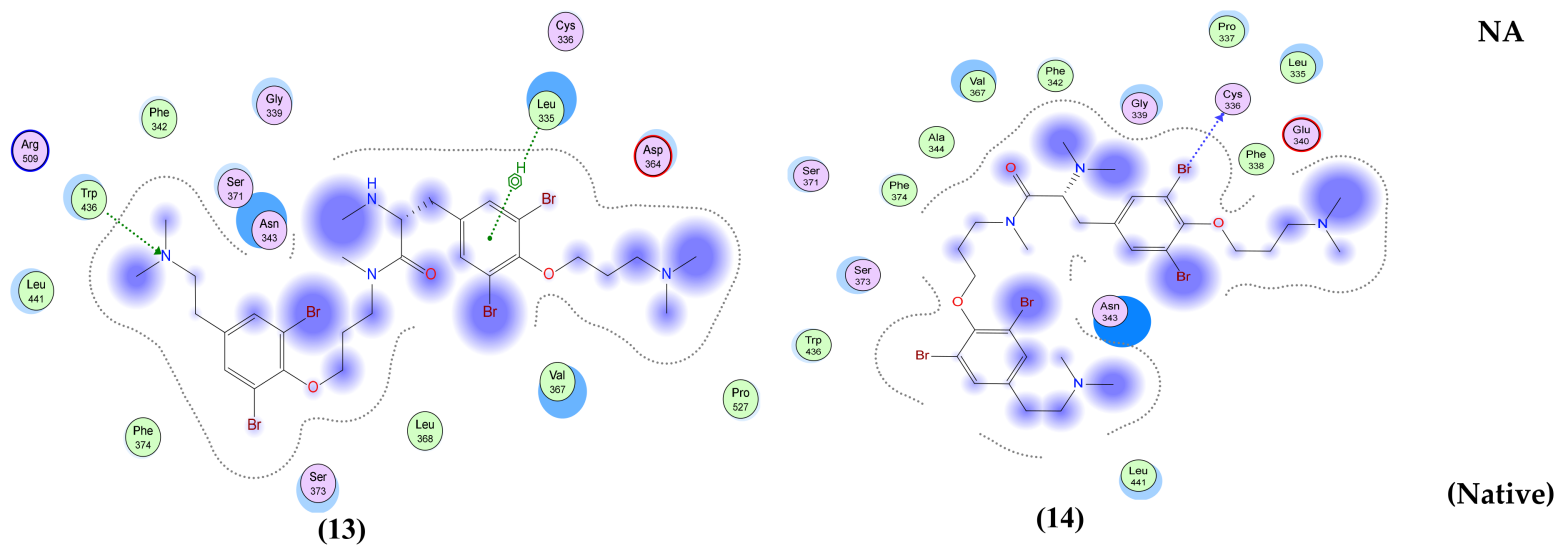
The 2D interactions of the 14 library compounds in addition to the native ligand with spike glycoprotein (PDB ID: 6VYB). NA: Non applicable.

Psammaplysin D (**5**) shows the strongest interactions with the spike glycoprotein, having 4 interactions with the active pocket: a H-bond donor between O101 of the ligand and Ser 373 in the pocket; two interactions as a H-bond acceptor; one between O45 of the ligand and Trp 436 of the receptor and another between N77 of the ligand and Val 367 in the pocket; and an H-pi interaction between C48 of the ligand and Trp 436 in the pocket. These interactions are greater than the native ligand, which only has one interaction with the active pocket: a H-bond donor between O6 of the ligand and Ser 343 in the pocket. Fistularin-3 (**3**) exhibits 4 interactions: one of them similar to the native ligand but with a better predicted binding affinity, and three more additional H-bond interactions. Two of these interactions are as a H-bond donor, one between O40 of the ligand and Asn 343 of the receptor and the other between O45 of the ligand and Val 367; and 2 H-bonds as an acceptor, one between O12 of the ligand and Gly 339 of the receptor and the other between O29 of the ligand and Ser 373 in the pocket. The 2D interactions for the fourteen library compounds in addition to the native ligand on spike glycoprotein are shown in Figure 5.

**Figure 6:**
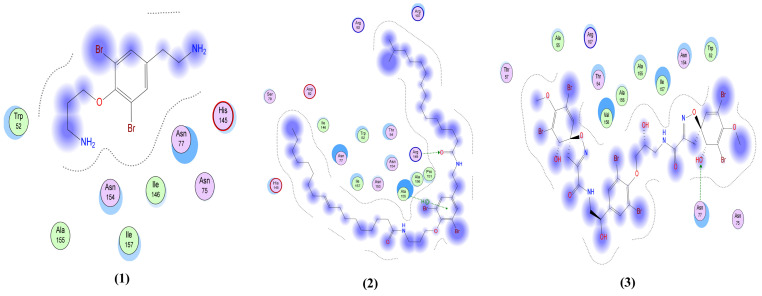

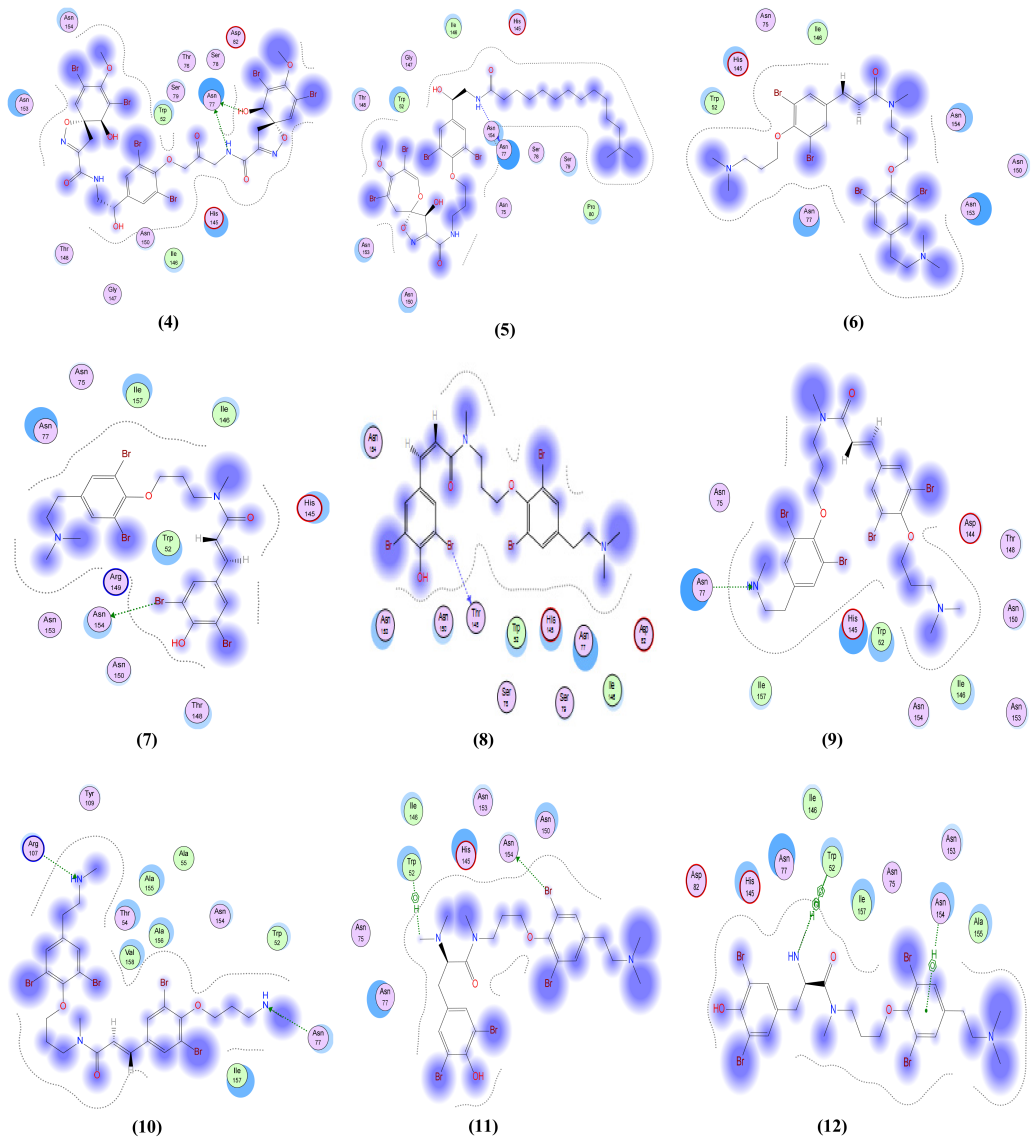

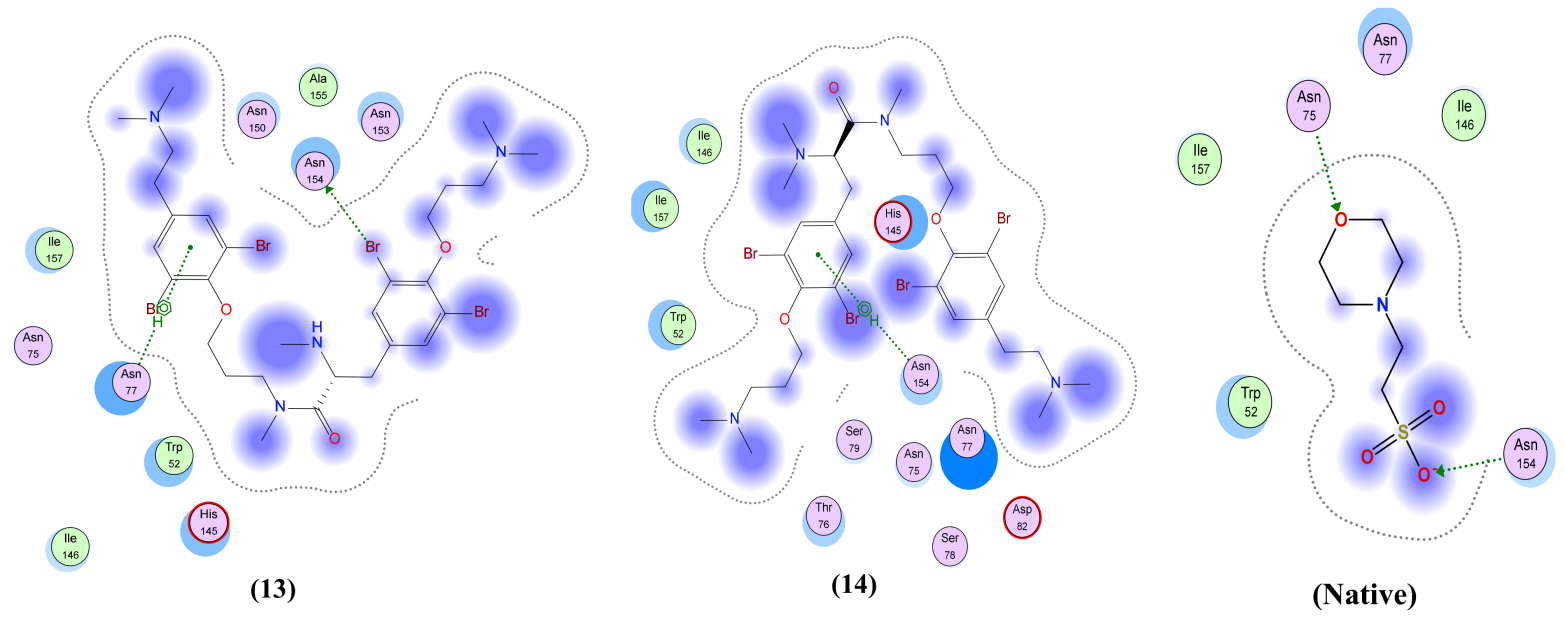
The 2D interactions of the 14 library compounds plus the native ligand with nucleocapsid phosphoprotein (PDB ID: 6VYO).

The native co-crystallized ligand formed two interactions with the pocket of the nucleocapsid phosphoprotein as H-bond acceptors with Asn 75 and Asn 154 in the pocket. As shown in Table 2 and Figure 6, compounds **3**, **4**, **11**, **12** and **13**, similar to the native ligand, form one or more similar interactions with the nucleocapsid phosphoprotein. However, these interactions for these compounds have higher predicted binding affinities (S-scores of −6.39, −6.84, −5.1, −5.74 and −5.69 Kcal/mol, respectively) compared to the native ligand (S-score = −4.44 Kcal/mol).

**Figure 7:**
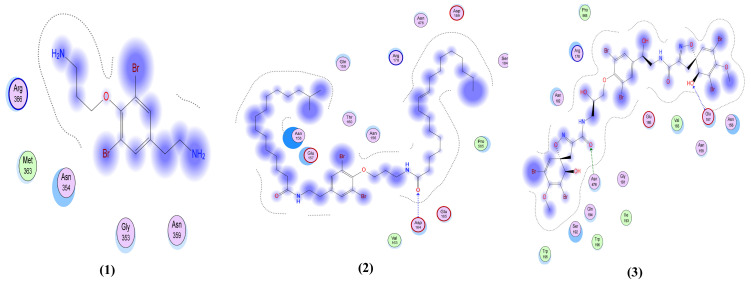

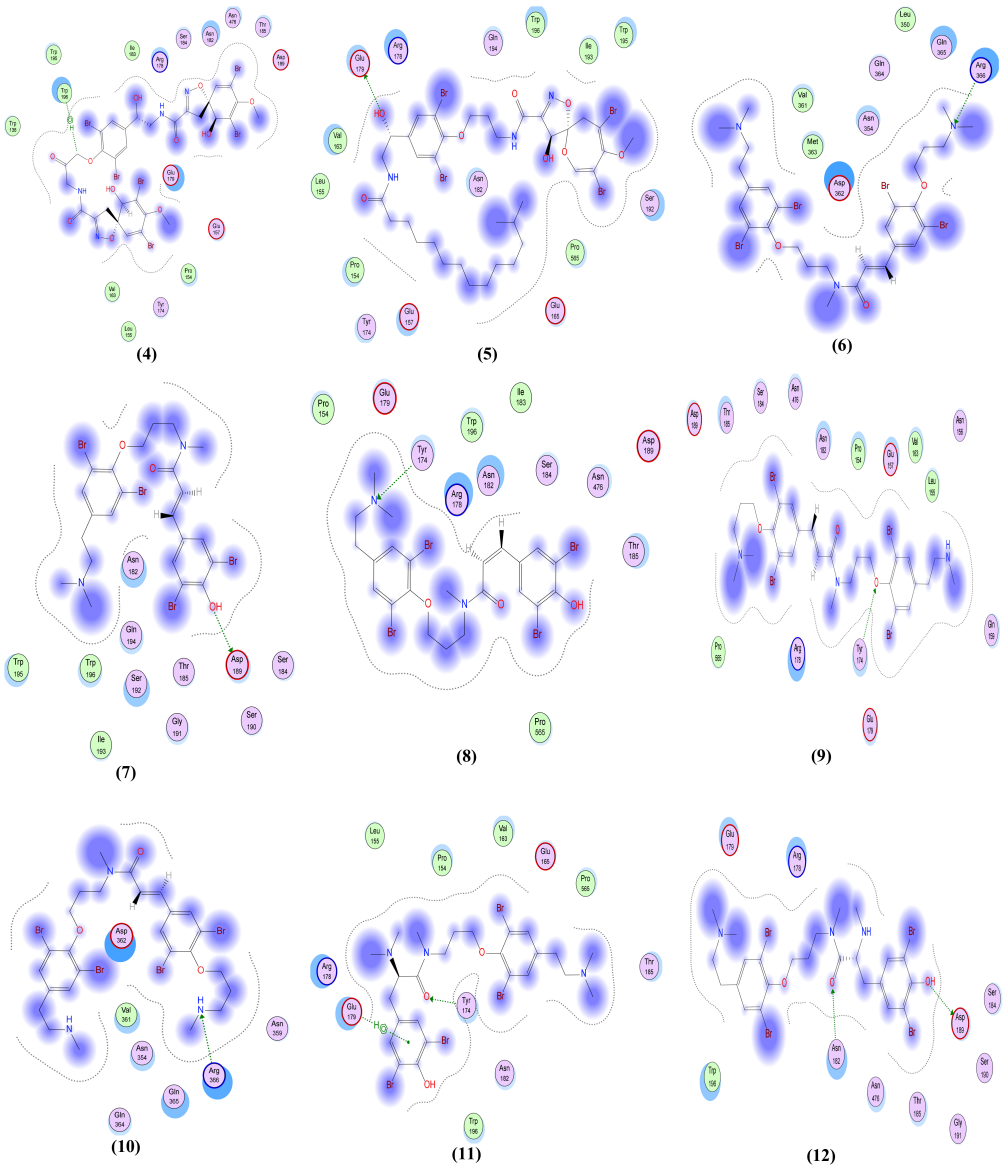

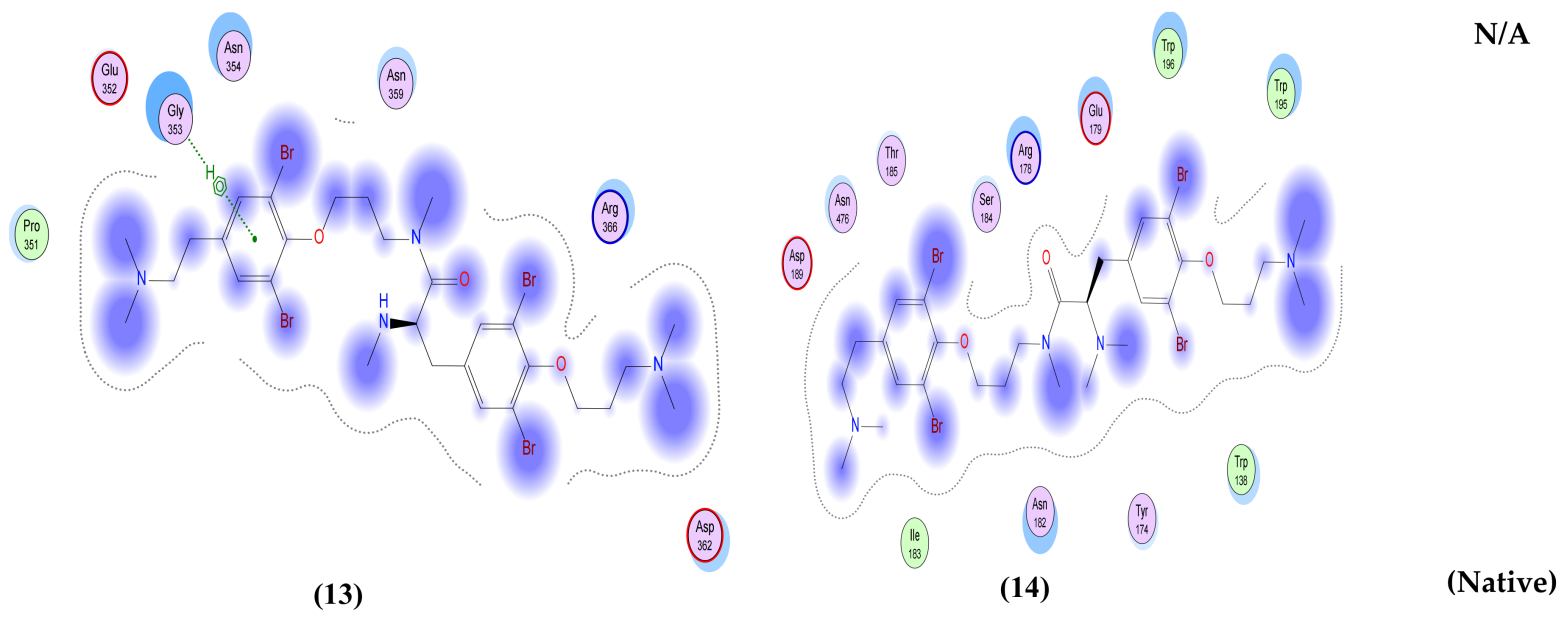
The 2D interactions between the 14 library compounds and the native ligand with membrane glycoprotein (PDB ID: 6M17).

Fistularin-3 (**3**) and anomoian C-D (**11–12**) showed the best interactions with the membrane glycoprotein as illustrated in Table 2 and Figure 7. Fistularin-3 (**3**) formed two H-bond acceptor interactions: one between ND2 of the ligand and Asn 476 of the receptor, and the other between O62 of the ligand and Glu 157 in the pocket. Anomoian C (**11**) displayed two interactions: one as a H-bond acceptor between O18 of the ligand and Tyr 174 in the pocket and the other as a pi-H interaction between the ligand’s 6-membered ring and Glu 179 in the pocket. Similarly, anomoian D (**12**) also formed two interactions, donating a H-bond by O1 of the ligand to Asp 189 in the pocket and accepting a H-bond by O18 of the ligand and Asn 182 in the pocket.

**Figure 8:**
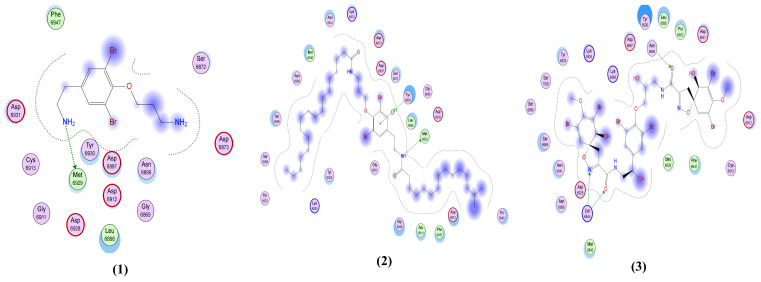

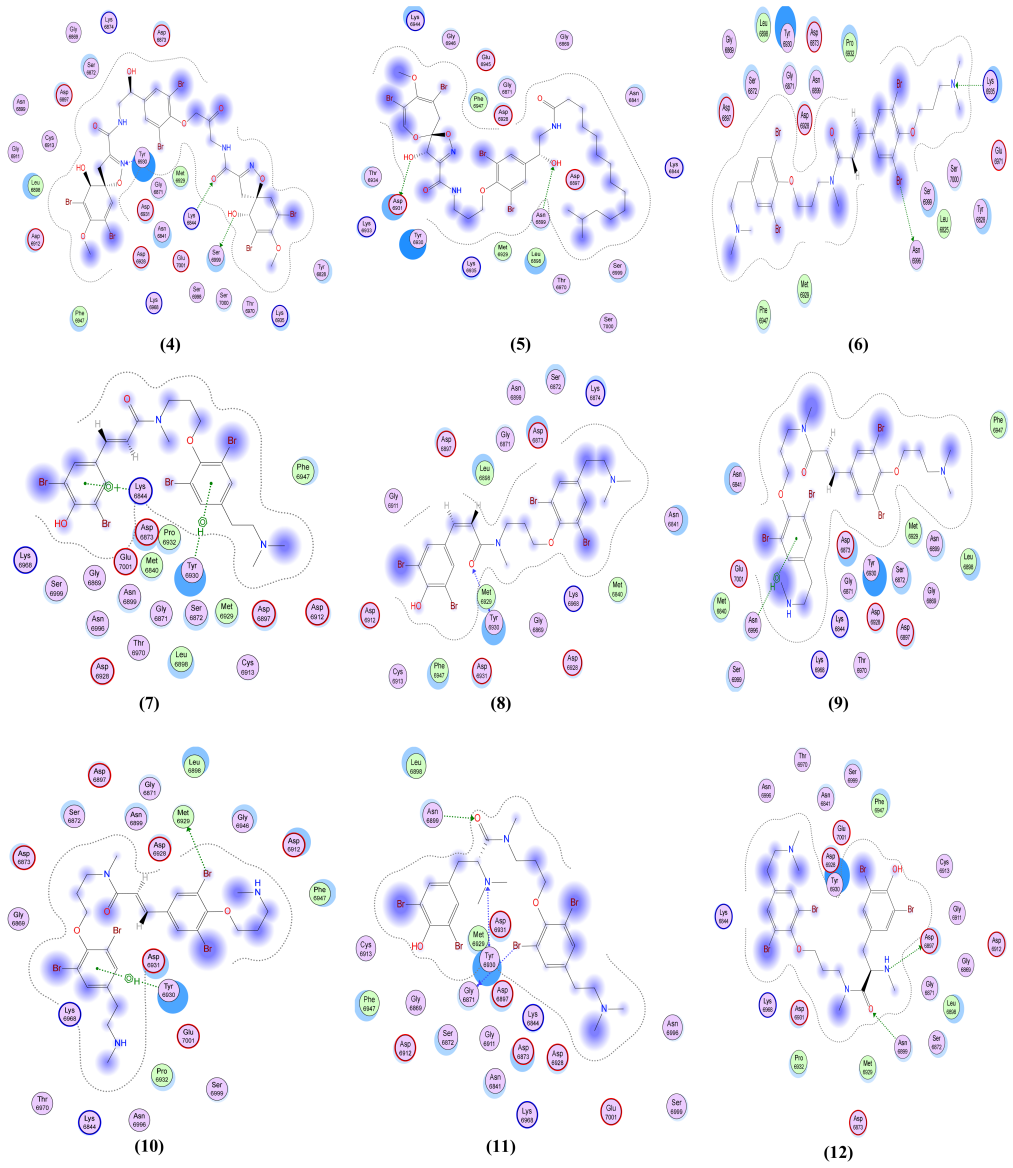

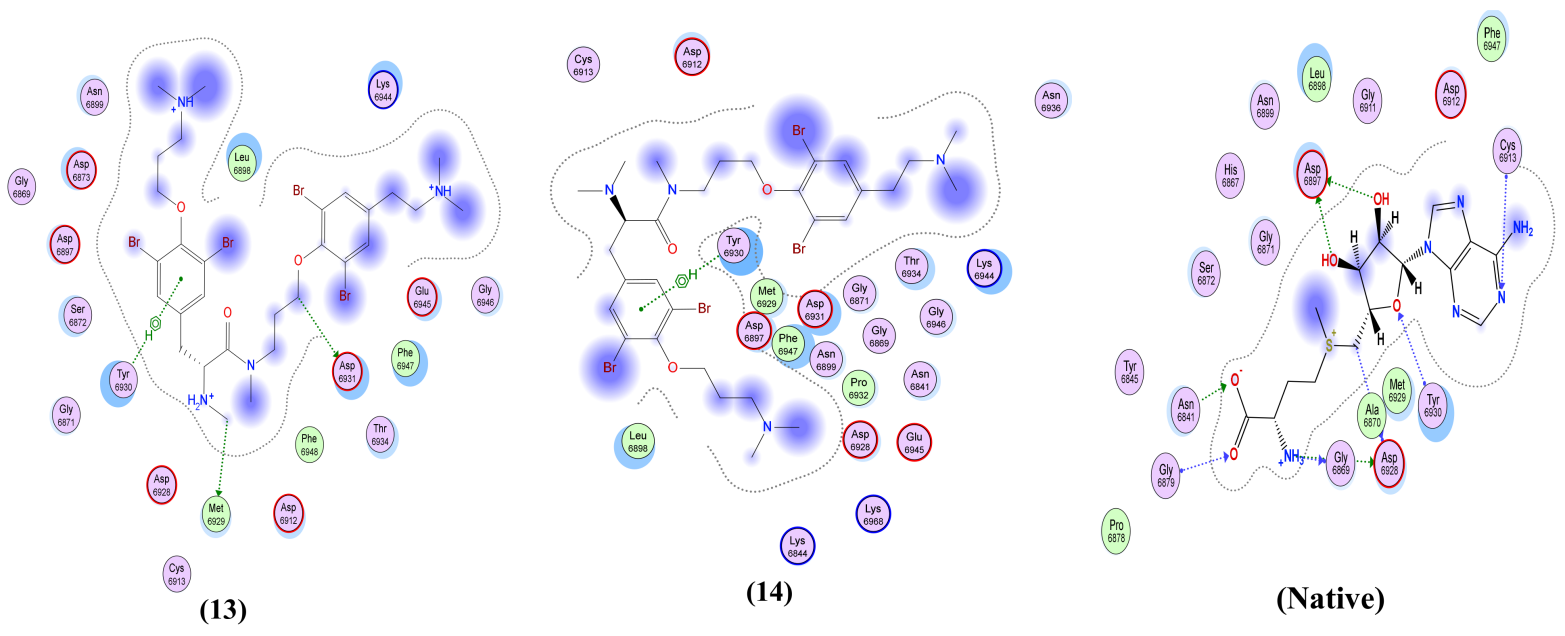
The 2D interactions of the 14 test compounds and the native ligand with the non-structural protein, nsp10 (PDB ID: 6W4H).

Compounds **3**, **4** and **11** showed the best interactions with the non-structural protein, nsp10, where all formed three interactions with the active pocket, as shown in Table 2 and Figure 8. Fistularin-3 (**3**) formed three H-bonds as an acceptor: one between O18 of the ligand and Asn 6899 of the receptor and the other two between O47 and N49 of the ligand and Lys 6844 in the pocket. In contrast, 11-ketofistularin (**4**) formed two H-bonds as an acceptor: one between N15 of the ligand and Tyr 6930 of the receptor and the other between O47 of the ligand and Lys 6844, as well as a third interaction as a H-bond donor by O62 of the ligand and Ser 6999 in the pocket. Similarly, anomoian C (**11**) had two H-bond acceptor interactions: one between O18 of the ligand and Asn 6899 of the receptor and the other between N60 of the ligand and Tyr 6930 in the pocket, and a H-bond donor between Br42 of the ligand and Gly 6871 in the pocket.

### 2.2. In Silico Prediction of Pharmacokinetics and Toxicity (ADME/Tox)

The pharmacokinetic properties of the 14 library compounds were calculated in silico using the SWISS-ADME and pkCSM online webtools. These results are shown in Table 3. Twelve of the fourteen compounds had very high logP values (>5) and, consequently, failed to comply with the Lipinski’s rule of five requirements in this respect; only compounds 1 and 3 had logP values <5, at 2.44 and 2.97, respectively. Other than **1** and **3**, all the other 12 compounds were poorly soluble, and thus had an unfavorable solubility. Compounds **2** and **5** were completely insoluble. Compound **1** had a high solubility and compound **3** was moderately soluble which agreed with their lipophilicity scores. Compound **1**, which showed effective pharmacokinetic properties, was the only compound predicted to be able to pass the BBB. Thus, this compound may possibly have side effects in the CNS.

Regarding metabolism, all compounds, except **1**, **3** and **4**, were potential substrates for CYP3A4 enzymes. On the other hand, no compound raised concerns with respect to medicinal chemistry parameters as possibly being pan-assay interference compounds (PAINS). All compounds also showed no potential cardiotoxicity as inhibitors of hERG1. In conclusion, compound **3** demonstrated the best combination of ADME/Tox properties among all 14 compounds.

### 2.3. Structure-Activity Relationships (SARs)

**Figure 9:**
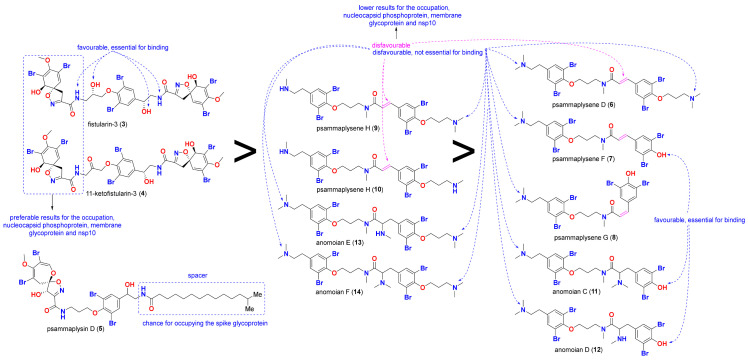
SARs of the studied compounds.

The SARs of this series of marine alkaloids, based on the results presented in Table 2, are summarized in Figure 9. It seems likely that the presence of the two terminal amines is essential for the interaction with M^pro^, while the primary terminal amines, in contrast, are not favorable to this interaction. Converting these terminal amines into amides connected to the unsaturated spiro [4,5]decane, as is the case with compound **3**, showed the greatest interaction with M^pro^, having the ability to occupy its four major pockets: S1, S2, S3 and S4.

The presence of the tertiary amines is also not favorable, as is the case for compounds **6**, **7**, **8** and **11**. For the interaction, at least one amine must be a secondary amine, as is the case for compounds **9**, **10** and **13**. However, attaching the amide group to a long saturated aliphatic chain, as in compound **5**, provides a better chance of occupying the spike glycoprotein.

Similar to M^pro^, the presence of the two amides connected to the unsaturated spiro[4,5]decane increases the predicted binding affinities of the nucleocapsid phosphoprotein, membrane glycoprotein, and nsp10, as clearly shown by compounds **3** and **4**. However, dissimilar to M^pro^, the presence of the two terminal amines is not favorable for binding to the nucleocapsid phosphoprotein, membrane glycoprotein, or nsp10, as is the case for compounds **11** and **12**. The presence of a terminal hydroxyl group on the other side of the compound increases the binding interactions in these compounds. The presence of alpha-beta unsaturated compounds, such as those in **6–10**, decreases binding ability.

### 2.4. Molecular Dynamics Simulation, Trajectory Post-Processing, Analysis, and MM/PBSA Calculations for Fistularin-3 *(**3**)*

**Figure 10:**
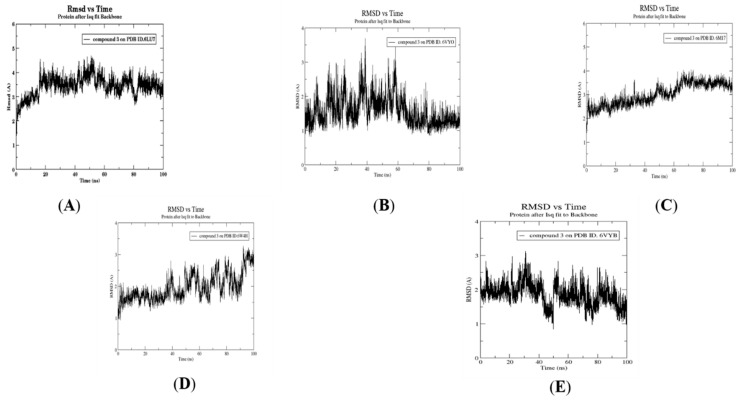
Dynamics of compound **3** bound to PDB ID: 6LU7 (**A**), 6VYO (**B**), 6M17 (**C**), 6W4H (**D**) and 6VYB (**E**), respectively. RMSD analysis of compound **3** against the five target proteins.

**Figure 11:**
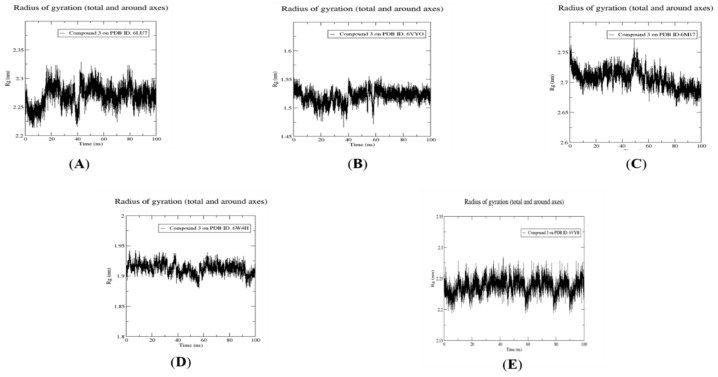
Dynamics of compound **3** bound to PDB ID: 6LU7 (**A**), 6VYO (**B**), 6M17 (**C**), 6W4H (**D**) and 6VYB (**E**), respectively. Radius of gyration analysis of compounds **3** against the five target proteins.

As compound **3** was the only compound that showed a combination between effective ADME/Tox properties and interactions with high S-scores in all five SARS-CoV-2 target proteins, it was selected for conducting a 100 ns MD simulation with the five target proteins. Figure 10 and Figure 11 show the RMSD fluctuations of protein–ligand complexes with respect to the initial structure, and the radius of gyration, respectively, for compound **3** with the five targets, respectively. This enabled the analysis of the stability of the simulated system throughout the 100 ns MD simulations. As expected, all complexes showed predicted, small RMSD fluctuations within only 2 Å, confirming their high stability throughout the whole simulation, where compound **3** showed the greatest stability with membrane glycoprotein (PDB ID: 6M17). Moreover, the radius of gyration was also consistent with high stability, with all fluctuations being within 0.05 nm.

**Figure 12:**
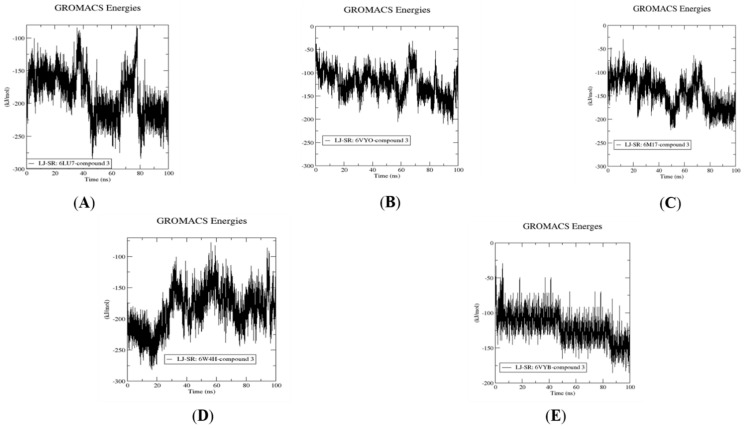
Dynamics of compound **3** bound to PDB ID: 6LU7 (**A**), 6VYO (**B**), s6M17 (**C**), 6W4H (**D**) and 6VYB (**E**), respectively. Binding energy using LJ-SR3.

Figure 12 displays protein-ligand interactions for the five complexes with compound **3** to quantify the strength of the interactions through computing non-bonded interaction energy. The least energy at −250 kJ/mol occurs when compound **3** binds to M^pro^ (PDB ID: 6LU7). Compound **3** has a higher stability with the other four SARS-CoV-2 proteins (~200 kJ/mol).

## 3. Materials and Methods

### 3.1. Preparation of the Screening Library

The MOL2 files for the five bromotyrosine derived compounds (**1–5**) that were recognized to have antiviral activities, shown in Figure 2, along with nine bromotyrosine derivatives (**6**–**14**) from the French Polynesian marine sponge, *Suberea ianthelliformis* [35], shown in Figure 3, were downloaded from the PubChem website (https://pubchem.ncbi.nlm.nih.gov/, accessed on 28 June 2021) and saved as mdb files using MOE v.2019.01.

### 3.2. Preparation of Protein Structures

The X-ray crystal structures for the five target proteins from SARS-CoV-2, including the main protease (M^Pro^; PDB ID: 6LU7), spike glycoprotein (PDB ID: 6VYB), nucleocapsid phosphoprotein (PDB ID: 6VYO), membrane glycoprotein (PDB ID: 6M17), and non-structural protein 10 (nsp10;PDB ID: 6W4H), were retrieved from the Protein Data Bank (http://www.pdb.org, accessed on 1 July 2021). Their resolutions were 2.16 Å, 3.20 Å, 1.70 Å, 2.90 Å, and 1.80 Å, respectively. All water molecules were removed from these crystal structures with only main-chain amino acids retained. An AMBER (AMBER10:EHT) force field was used for the energy minimization of these five X-ray crystal structures using parameters suitable for proteins and nucleic acids (ff10) and small molecules (EHT). Protons were added by employing the 3D protonation feature in MOE v.2019.01; Asn, Gln and His flips were allowed during 3D protonation. Complexes were then refined to a RMS gradient of 0.1 Kcal/mol/Å.

### 3.3. Re-Docking of the Co-Crystallized Ligand and Docking of Screening Library

The fourteen compounds in our screening library (**1**–**14**) were docked with the five SARS-CoV-2 target proteins, main protease (M^Pro^; PDB ID: 6lu7), spike glycoprotein (PDB ID: 6VYB), nucleocapsid phosphoprotein (PDB ID: 6VYO), membrane glycoprotein (PDB ID: 6M17), and non-structural protein 10 (nsp10; PDB ID: 6W4H) using MOE v.2019.01. In addition, re-docking the target proteins’ co-crystallized ligands was performed for validation purposes except for 6M17. Docking validation figures are included in the Supplementary Materials (Table S1). For docking scoring, triangle matcher placement was used with the first rescoring function set to London dG and GBVI/WSA dG used as the second rescoring function. Docking was ultimately refined with a force field retaining 30 docked structures for each compound. Root Mean Square Deviation (RMSD) values between the docked conformation and the reference conformation, presented in Å, was utilized to validate docking performances [38].

### 3.4. In Silico Prediction of Pharmacokinetics and Toxicity

The pharmacokinetic properties of the fourteen compounds in our screening library were calculated using the SWISS-ADME webtool (https://www.swissadme.ch, accessed on 28 June 2021). The properties predicted here were lipophilicity, reported as Log Po/w (WLOGP); water solubility class; and Blood–brain barrier (BBB) penetration, in addition to medicinal chemistry parameters employing pan-assay interference alerts (PAINS) [39,40]. Additionally, the potential toxicity profiles of these molecules were predicted using the pkCSM online webtool (http://biosig.unimelb.edu.au/pkcsm/prediction, accessed on 28 June 2021) to predict the safety of these small molecules upon ingestion in human and animal models, with respect to toxicological effects on hERG-I inhibition [41].

### 3.5. Molecular Dynamics Simulation for Compound ***3***

Compound **3**, Fistularin-3, displayed the best binding interactions and free energies with the five SARS-CoV-2 target proteins among the fourteen library compounds investigated. It also showed the best pharmacokinetics properties. Accordingly, it was subjected to 100 ns molecular dynamics investigation against the five SARS-CoV-2 target proteins. MD simulations were performed using the GROMACS 2021 software package with the CHARMM36 force field used for protein topology preparation and the official CHARMM General Force Field server (CGenFF) used for ligand topology preparation. The solvation method used was a dodecahedron box of common simple point charge (SPC) water model with explicit solvent periodic boundary conditions. Charge neutralization using sodium and chloride ions was performed for the five solvated complexes. These systems were subjected to energy minimization to resolve steric clashes or inappropriate geometry employing the steepest descent method of 5000 steps. System equilibration was also set to ensure a reasonable starting structure using NVT and equilibration under constant number of particles, volume, and temperature (NVT) for 100 ps using a Berendsen thermostat [42]. Then, re-equilibration was performed for another 100 ps under constant pressure (Isothermal-isobaric (NPT) ensemble) using the Parrinello–Rahman barostat using a time step of 2 fs for each equilibration round [43]. Finally, an MD production phase was performed for 100 ns using a time step of 2 fs at a constant temperature of 300 K and constant pressure of 1 atm. Simulation results were analyzed using Visual Molecular Dynamics (VMD) software, ver.1.9.3 [44].

### 3.6. Post MD Analysis, Trajectory Post-Processing and MM/PBSA Calculations

After determining the trajectories of the five complexes resulting from the MD simulation of compound **3**, the complexes were re-centered and rewrapped within unit cells using the trjconv function of GROMACS. The stabilities of trajectories were then determined throughout the 100 ns simulation using the radius of gyration and the root-mean-square deviation (RMSD) of the protein backbone referenced to its initial position at 10 ps intervals. Lastly, g_mmpbsa was employed using Molecular Mechanics/Generalized Born Surface Area (MM/GBSA) binding free energy [45] to calculate relative binding free energies according to the following equation:

(1) $$\Delta G_{bind}=G_{complex}-G_{protein}-G_{ligand}$$

(2) $$\Delta G_{bind}=\Delta E_{gas}+\Delta G_{solvation}-T\Delta S\;$$

(3) $$\Delta E_{gas}=E_{int}+E_{vdw}+E_{elec}$$

(4) $$E_{int}=E_{bond}+E_{angle}+E_{torsion}\;$$

(5) $$G_{solvation,\;GB}=G_{GB}+G_{nonpolar,\;solvation}-G_{ligand}\;$$

(6) $$\Delta G_{nonpolar}=\gamma SASA+\beta$$

## 4. Conclusions

Fourteen structurally diverse brominated tyrosine alkaloids were comprehensively explored for their virtual antiviral potentials against five SARS-CoV-2 proteins. Among the tested compounds, the polybrominated alkaloid, fistularin-3 (**3**), displayed the best docking scores with predicted binding affinities (S-score = −7.78, −7.65, −6.39, −6.28, −8.84 Kcal/mol) for main protease (M^pro^) (PDB ID: 6lu7), spike glycoprotein (PDB ID: 6VYB), nucleocapsid phosphoprotein (PDB ID: 6VYO), membrane glycoprotein (PDB ID: 6M17), and non-structural protein 10 (nsp10) (PDB ID: 6W4H), respectively, where it formed better interactions with the protein pockets than the native interaction. This was supported by very stable molecular dynamics simulations. In addition, it was found that compound **3** structurally complied with the previously reported structural and pharmacophoric requirements for efficient bio-target binding [37,46]. Considering the feasibility of synthesizing structurally related compounds/congeners of compound **3** [47,48,49,50,51], it seems reasonable to test an expanded library based on the structure of this compound. This may provide rich novel candidates that function as COVID-19 antiviral compounds.

## Figures and Tables

**Table 1:** Docking S-score (Kcal/mol) for screening library compounds compared to those of co-crystallized native ligands with the five SARS-CoV-2 target proteins.

Compound	Main Protease (PDB ID: 6lu7)	Spike Glycoprotein (PDB ID: 6VYB)	Nucleocapsid Phosphoprotein (PDB ID: 6VYO)	Membrane Glycoprotein (PDB ID: 6M17)	Non-Structural Protein 10 (nsp10) (PDB ID: 6W4H)
Moloka′Iamine (**1**)	−5.97	−5.14	−4.33	−3.44	−5.38
Mololipids (**2**)	−7.92	−7.14	−7.04	−6.24	−9.37
Fistularin-3 (**3**)	−7.78	−7.65	−6.39	−6.28	−8.84
11-ketofistularin-3 (**4**)	−8.02	−6.77	−6.84	−5.97	−9.77
Psammaplysin D (**5**)	−8.01	−7.09	−6.91	−6.06	−9.24
Psammaplysene D (**6**)	−7.46	−6.71	−5.74	−4.98	−7.79
Psammaplysene F (**7**)	−7.11	−6.60	−5.48	−5.37	−7.86
Psammaplysene G (**8**)	−7.62	−6.71	−5.29	−5.24	−7.06
Psammaplysene H (**9**)	−7.36	−7.03	−5.96	−5.59	−7.67
Psammaplysene I (**10**)	−7.80	−6.16	−5.47	−5.97	−7.21
Anomoian C (**11**)	−7.38	−6.70	−5.10	−5.07	−7.25
Anomoian D (**12**)	−7.30	−6.47	−5.74	−4.58	−7.48
Anomoian E (**13**)	−8.54	−7.29	−5.69	−4.56	−8.41
Anomoian F (**14**)	−7.72	−6.46	−6.01	−5.56	−7.73
Native co-crystallized ligand	−8.25	−4.55	−4.44	NA	−9.43

NA: Non applicable.

**Table 2:** The 2D interactions of compounds in the screening library compared to those of native co-crystallized ligands with the five SARS-CoV-2 target proteins.

Compound	Main Protease (M^Pro^) (PDB ID: 6lu7)	Spike Glycoprotein (PDB ID: 6VYB)	Nucleocapsid Phosphoprotein (PDB ID: 6VYO)	Membrane Glycoprotein (PDB ID: 6M17)	Non-Structural Protein 10 (nsp10) (PDB ID: 6W4H)
Moloka′Iamine (**1**)	One interaction: -H-bond acceptor between N5 of the ligand and His 163 in the pocket with distance 3.47 Å and energy scores of −1.8 Kcal/mol.	One interaction: -Pi-H interaction between the ligand’s 6-membered ring and Gly 339 in the pocket with distance 3.71 Å and energy scores of −0.8 Kcal/mol.	Only hydrophobic interaction with the pocket	Only hydrophobic interaction with the pocket	One interaction: -H-bond donor between N4 of the ligand and Met 6929 in the pocket with distance 3.83 Å and energy scores of −0.9 Kcal/mol.
Mololipids (**2**)	Only hydrophobic interaction with the pocket	One interaction: -H-bond acceptor between O52 of the ligand and Gly 339 in the pocket with distance 3.27 Å and energy scores of −1.0 Kcal/mol.	Two interactions: -H-bond acceptor between O83 of the ligand and Arg 149 in the pocket with distance 2.9 Å and energy scores of −4.7 Kcal/mol. A -Pi-H interaction between the ligand’s 6-membered ring and Ala 155 in the pocket with distance 3.83 Å and energy scores of −1.0 Kcal/mol.	One interaction: -H-bond acceptor between O52 of the ligand and Asp 164 in the pocket with distance 3.22 Å and energy scores of −2.7 Kcal/mol.	Two interactions: -H-bond donor between N80 of the ligand and Met 6929 in the pocket with distance 4.41 Å and energy scores of −0.9 Kcal/mol. A -Pi-H interaction between the ligand’s 6-membered ring and Tyr 6930 in the pocket with distance 4.33 Å and energy scores of −1.0 Kcal/mol.
Fistularin-3 (**3**)	Three interactions: -2 H-bond donors between N44 and O71 of the ligand and Glu 166 in the pocket with distances 2.95 Å and 2.97 Å, respectively, and with energy scores of −4.6 Kcal/mol and −0.9 Kcal/mol, respectively. A -Pi-H interaction between the ligand’s 6-membered ring and Glu 166 in the pocket with distance 4.48 Å and energy score of −0.7 Kcal/mol.	Four interactions: -2 H-bond donors, one between O40 of the ligand and Asn 343 of the receptor and the other between O45 of the ligand and Val 367 in the pocket, with distances 2.65 Å and 2.63 Å, respectively, and with energy scores of −2.3 Kcal/mol and −2.2 Kcal/mol, respectively. There are -2 H-bond acceptors, one between O12 of the ligand and Gly 339 of the receptor and the other one between O29 of the ligand and Ser 373 in the pocket, with distances 2.91 Å and 2.51 Å, respectively, and with energy scores of −4.8 Kcal/mol and Ȓ0.8 Kcal/mol, respectively.	One interaction: -H-bond acceptor between O80 of the ligand and Asn 77 in the pocket with distance 3.36 Å and energy score of −0.6 Kcal/mol.	Two interactions: -2 H-bond acceptors, one between ND2 of the ligand and Asn 476 of the receptor and the other one between O62 of the ligand and Glu 157 in the pocket, with distances 3.07 Å and 3.32 Å, respectively, and with energy scores of −0.8 Kcal/mol and −0.7 Kcal/mol, respectively.	Three interactions: -3 H-bond acceptors, one between O18 of the ligand and Asn 6899 of the receptor and the other 2 interactions between O47 and N49 of the ligand and Lys 6844 in the pocket, with distances 3.15 Å, 2.92 Å and 3.87 Å, respectively, and with energy scores of −2.3 Kcal/mol, −1.1 Kcal/mol and −0.9 Kcal/mol, respectively.
11-ketofistularin-3 (**4**)	Two interactions: -H-bond donor, one between O78 of the ligand and Glu 166 in the pocket, with distances 3.15 Å and with energy score of −1.1 Kcal/mol. A -H-bond acceptor between O40 of the ligand and Gly 143 in the pocket with distance 3.41 Å and energy score of −1.3 Kcal/mol.	One interaction: -H-bond acceptor between O47 of the ligand and Asn 343 in the pocket with distance 2.79 Å and energy scores of −4.6 Kcal/mol.	Two interactions: -2 H-bond donors between N44 and O62 of the ligand and Asn 77 in the pocket with distances 3.08 Å and 2.90 Å, respectively, and with energy scores of −1.5 Kcal/mol and −0.7 Kcal/mol, respectively.	One interaction: -H-Pi interaction between C36 of the ligand and Trp 196 in the pocket with distance 4.37 Å and energy score of −1.2 Kcal/mol.	Three interactions: -H-bond donor between O62 of the ligand and Ser 6999 in the pocket with distance 2.74 Å and energy score of −1.0 Kcal/mol. There are -2 H-bond acceptors: one between N15 of the ligand and Tyr 6930 of the receptor and the other one between O47 of the ligand and Lys 6844 in the pocket, with distances 2.87 Å and 3.14 Å, respectively, and with energy scores of −1.2 Kcal/mol and −5.5 Kcal/mol, respectively.
Psammaplysin D (**5**)	Two interactions: -2 H-bond donors, one between N46 of the ligand and Glu 166 of the receptor and the other one between O82 of the ligand and Ser 46 in the pocket, with distances 3.05 Å and 2.96 Å, respectively, and with energy scores of −1.8 Kcal/mol and −1.6 Kcal/mol, respectively.	Four interactions: -H-bond donor between O101 of the ligand and Ser 373 in the pocket with distance 3.1 Å and energy score of −0.9 Kcal/mol. There are -2 H-bond acceptors, one between O45 of the ligand and Trp 436 of the receptor and the other one between N77 of the ligand and Val 367 in the pocket, with distances 3.11 Å and 3.5 Å, respectively, and with energy scores of −1.7 Kcal/mol and Ȓ0.9 Kcal/mol, respectively. A -H-Pi interaction between C48 of the ligand and Trp 436 in the pocket with distance 4.34 Å and energy score of −0.8 Kcal/mol.	One interaction: -H-bond donor between N46 of the ligand and Asn 77 in the pocket with distances 3.38 Å and with energy score of −1.0 Kcal/mol.	One interaction: -H-bond donor between O101 of the ligand and Glu 179 in the pocket with distances 2.95 Å and with energy score of −2.6 Kcal/mol.	Two interactions: -H-bond donor between O82 of the ligand and Asp 6931 in the pocket with distances 3.10 Å and with energy score of −0.7 Kcal/mol. A -H-bond acceptor between O101 of the ligand and Asn 6899 in the pocket with distance 3.05 Å and energy score of −2.1 Kcal/mol.
Psammaplysene D (**6**)	Only hydrophobic interaction with the pocket	One interaction: -Pi-H interaction between the ligand’s 6-membered ring and Gly 339 in the pocket with distance 4.29 Å and energy scores of −0.8 Kcal/mol.	Only hydrophobic interaction with the pocket	One interaction: -H-bond acceptor between N5 of the ligand and Arg 366 in the pocket with distance 3.32 Å and energy score of −4.0 Kcal/mol.	Two interactions: -H-bond donor between Br75 of the ligand and Asn 6996 in the pocket with distances 3.89 Å and with energy score of −1.0 Kcal/mol. A -H-bond acceptor between NZ of the ligand and Lys 6935 in the pocket with distance 3.13 Å and energy score of −6.5 Kcal/mol.
Psammaplysene F (**7**)	Only hydrophobic interaction with the pocket	Two interactions: -H-bond donor between Br41 of the ligand and Phe 342 in the pocket with distance 3.5 Å and energy score of −0.7 Kcal/mol. A -H-bond acceptor between O17 of the ligand and Asn 343 in the pocket with distance 3.23 Å and energy score of −0.8 Kcal/mol.	One interaction: -H-bond donor between Br58 of the ligand and Asn 154 in the pocket with distances 3.63 Å and with energy score of −0.6 Kcal/mol.	One interaction: -H-bond donor between O1 of the ligand and Asp 189 in the pocket with distances 2.97 Å and with energy score of −2.2 Kcal/mol.	Two interactions: -Pi-cation interaction between the ligand’s 6-membered ring and Lys 6844 in the pocket with distance 3.96 Å and energy score of −1.2 Kcal/mol. A -Pi-H interaction between the ligand’s 6-membered ring and Tyr 6930 in the pocket with distance 4.49 Å and energy score of −1.0 Kcal/mol.
Psammaplysene G (**8**)	One interaction: -H-bond donor between O48 of the ligand and Thr 190 in the pocket with distance 3.01 Å and energy score of −1.0 Kcal/mol.	One interaction: -H-bond donor between Br58 of the ligand and Phe 342 in the pocket with distance 3.79 Å and energy score of −0.4 Kcal/mol.	One interaction: -H-bond donor between Br58 of the ligand and Thr 148 in the pocket with distances 3.51 Å and with energy score of −1.6 Kcal/mol.	One interaction: -H-bond acceptor between N37 of the ligand and Tyr 174 in the pocket with distance 3.29 Å and energy score of −1.4 Kcal/mol.	One interaction: -H-bond acceptor between O6 of the ligand and Tyr 6930 in the pocket with distance 3.28 Å and energy score of −1.2 Kcal/mol.
Psammaplysene H (**9**)	Two interactions: -H-bond donor between Br72 of the ligand and Thr 45 in the pocket with distances 3.68 Å and with energy score of −1.1 Kcal/mol. A -Pi-H interaction between the ligand’s 6-membered ring and Glu 166 in the pocket with distance 4.63 Å and energy score of −0.6 Kcal/mol.	One interaction: -Pi-H interaction between the ligand’s 6-membered ring and Gly 339 in the pocket with distance 3.81 Å and energy score of −0.6 Kcal/mol.	One interaction: -H-bond acceptor between N65 of the ligand and Asn 77 in the pocket with distance 3.56 Å and energy score of −0.9 Kcal/mol.	One interaction: -H-bond acceptor between O49 of the ligand and Tyr 174 in the pocket with distance 3.05 Å and energy score of −1.0 Kcal/mol.	One interaction: -Pi-H interaction between the ligand’s 6-membered ring and Asn 6996 in the pocket with distance 4.52 Å and energy score of −0.6 Kcal/mol.
Psammaplysene I (**10**)	Four interactions: -3 H-bond donors, one between N62 of the ligand and Thr 190 of the receptor, the second one between Br68 of the ligand and Glu 166 in the pocket, and the last one between Br69 of the ligand and Cys 145 in the pocket with distances 2.93 Å, 3.52 Å and 3.77 Å, respectively, and with energy scores of −0.9 Kcal/mol, −1.9 Kcal/mol and −0.9 Kcal/mol, respectively. A -H-pi interaction between C7 of the ligand and His 41 in the pocket with distance 4.10 Å and energy score of −0.7 Kcal/mol.	One interaction: -H-bond donor between Br68 of the ligand and Asn 437 in the pocket with distance 3.82 Å and energy score of −0.8 Kcal/mol.	Two interactions: -2 H-bond acceptors, one between N5 of the ligand and Asn 77 of the receptor and the other one between N62 of the ligand and Arg 107 in the pocket, with distances 3.28 Å and 3.49 Å, respectively, and with energy scores of −1.5 Kcal/mol and −2.6 Kcal/mol, respectively.	One interaction: -H-bond acceptor between N5 of the ligand and Arg 366 in the pocket with distance 3.55 Å and energy score of −1.4 Kcal/mol.	Two interactions: -H-bond donor between Br69 of the ligand and met 6929 in the pocket with distance 4.05 Å and energy score of −0.6 Kcal/mol. A -Pi-H interaction between the ligand’s 6-membered ring and Tyr 6930 in the pocket with distance 4.15 Å and energy score of −0.8 Kcal/mol.
Anomoian C (**11**)	One interaction: -H-bond acceptor between O18 of the ligand and Glu 166 in the pocket with distance 2.84 Å and energy score of −1.2 Kcal/mol.	One interaction: -H-bond acceptor between N49 of the ligand and Trp 436 in the pocket with distance 3.17 Å and energy score of −1.3 Kcal/mol.	Two interactions: -H-bond donor between Br58 of the ligand and Asn 154 in the pocket with distances 3.49 Å and with energy score of −1.2 Kcal/mol. An -H-Pi interaction between C61 of the ligand and Trp 52 in the pocket with distance 4.05 Å and energy scores of −0.8 Kcal/mol.	Two interactions: -H-bond acceptor between O18 of the ligand and Tyr 174 in the pocket with distance 3.08 Å and energy score of −1.4 Kcal/mol. A -pi-H interaction between the ligand’s 6-membered ring and Glu 179 in the pocket with distance 4.35 Å and energy score of −0.7 Kcal/mol.	Three interactions: -H-bond donor between Br42 of the ligand and Gly 6871 in the pocket with distances 3.48 Å and with energy score of −1.4 Kcal/mol. There are -2 H-bond acceptors, one between O18 of the ligand and Asn 6899 of the receptor and the other one between N60 of the ligand and Tyr 6930 in the pocket, with distances 2.78 Å and 3.43 Å, respectively, and with energy scores of −2.6 Kcal/mol and −1.7 Kcal/mol, respectively.
Anomoian D (**12**)	Two interactions: -H-bond donor between Br11 of the ligand and Thr 45 in the pocket with distances 3.58 Å and with energy scores of −1.2 Kcal/mol. A -Pi-H interaction between the ligand’s 6-membered ring and Glu 166 in the pocket with distance 4.41 Å and energy score of −1.1 Kcal/mol.	Two interactions: -H-bond acceptor between O18 of the ligand and Asn 343 in the pocket with distance 3.14 Å and energy score of −1.0 Kcal/mol. A -Pi-H interaction between the ligand’s 6-membered ring and Gly 339 in the pocket with distance 3.66 Å and energy score of −1.0 Kcal/mol.	Three interactions: -2 H-pi interaction between N60 and C62 of the ligand and Trp 52 in the pocket with distances 3.86 Å and 3.80 Å, respectively, and energy scores of −0.6 Kcal/mol and −0.9 Kcal/mol, respectively. A -Pi-H interaction between the ligand’s 6-membered ring and Asn 154 in the pocket with distance 3.62 Å and energy score of −1.0 Kcal/mol.	Two interactions: -H-bond donor between O1 of the ligand and Asp 189 in the pocket with distance 3.06 Å and energy score of −2.3 Kcal/mol. A -H-bond acceptor between O18 of the ligand and Asn 182 in the pocket with distance 2.99 Å and energy score of −2.6 Kcal/mol.	Two interactions: -H-bond donor between N60 of the ligand and Asp 6897 in the pocket with distance 3.29 Å and energy score of −0.7 Kcal/mol. A -H-bond acceptor between O18 of the ligand and Asn 6899 in the pocket with distance 3.16 Å and energy score of −1.7 Kcal/mol.
Anomoian E (**13**)	Two interactions: -H-bond donor, one between Br37 of the ligand and Asn 142 of the receptor, with distance 3.62 Å and with energy score of −0.8 Kcal/mol. A -Pi-H interaction between the ligand’s 6-membered ring and Glu 166 in the pocket with distance 4.52 Å and energy score of −1.5 Kcal/mol.	Two interactions: -H-bond acceptor between N66 of the ligand and Trp 436 in the pocket with distance 3.13 Å and energy score of −1.8 Kcal/mol. A -Pi-H interaction between the ligand’s 6-membered ring and Leu 335 in the pocket with distance 4.07 Å and energy score of −0.8 Kcal/mol.	Two interactions: -H-bond donor between Br28 of the ligand and Asn 154 in the pocket with distance 3.50 Å and energy score of −3.0 Kcal/mol. A -pi-H interaction between the ligand’s 6-membered ring and Asn 77 in the pocket with distance 3.77 Å and energy score of −1.1 Kcal/mol.	One interaction: -Pi-H interaction between the ligand’s 6-membered ring and Gly 353 in the pocket with distance 4.01 Å and energy score of −1.1 Kcal/mol.	Two interactions: -H-bond donor between C47 of the ligand and Asp 6931 in the pocket with distance 3.42 Å and energy score of −0.7 Kcal/mol. A -Pi-H interaction between the ligand’s 6-membered ring and Tyr 6930 in the pocket with distance 4.52 Å and energy score of −1.2 Kcal/mol.
Anomoian F (**14**)	Two interactions: -H-bond acceptor between O35 of the ligand and Cys 145 in the protein pocket with distance 3.36 Å and energy score of −1.1 Kcal/mol. A -Pi-H interaction between the ligand’s 6-membered ring and Glu 166 in the pocket with distance 4.11 Å and energy score of −0.6 Kcal/mol.	One interaction: -H-bond donor between Br28 of the ligand and Cys 336 in the pocket with distance 3.57 Å and energy score of −1.4 Kcal/mol.	One interaction: -Pi-H interaction between the ligand’s 6-membered ring and Asn 154 in the pocket with distance 4.24 Å and energy score of −1.2 Kcal/mol.	Only hydrophobic interaction with the pocket	Two interactions: -H-bond donor between Br28 of the ligand and Gly 6869 in the pocket with distance 3.79 Å and energy score of Ȓ0.5 Kcal/mol. A -Pi-H interaction between the ligand’s 6-membered ring and Tyr 6930 in the pocket with distance 4.72 Å and energy score of −0.7 Kcal/mol.
Native co-crystallized ligand	Three interactions: -2 H-bond donors between N1 and N18 of the ligand and Glu 166 in the pocket with distances 3.08 Å and 3.3 Å, respectively, and with energy scores of −1.1 Kcal/mol and −1.6 Kcal/mol, respectively. A -Pi-H interaction between the ligand’s 6-membered ring and Thr 26 in the pocket with distance 4.29 Å and energy score of −0.5 Kcal/mol.	One interaction: -H-bond donor between O6 of the ligand and Asn 343 in the pocket with distance 2.9 Å and energy score of −0.9 Kcal/mol.	Two interactions: -2 H-bond acceptors, one between O1 of the ligand and Asn 75 of the receptor and the other between O1S of the ligand and Asn 154 in the pocket, with distances 2.86 Å and 2.41 Å, respectively, and with energy scores of −3.0 Kcal/mol and −14.2 Kcal/mol, respectively.	NA	Two interactions: -6 H-bond donors, one between N1 of the ligand and Gly 6869 of the receptor, the second between N1 of the ligand and Asp 6928 in the pocket, the third between C5’ of the ligand and Asp 6928 in the pocket, the fourth between O3’ of the ligand and Asp 6897 in the pocket, the fifth between O2’ of the ligand and Asp 6897, and the sixth between N6 of the ligand and Asp 6912 in the pocket, with distances 2.70 Å, 2.69 Å, 3.40 Å, 3.04 Å, 2.84 Å and 3.01 Å, respectively, and with energy scores of −12.5 Kcal/mol, −12.4 Kcal/mol, Ȓ0.8 Kcal/mol, −3.1 Kcal/mol, −4.5 Kcal/mol and −1.7 Kcal/mol, respectively. There are -5 H-bond acceptors: the first between O8 of the ligand and Gly 6879 of the receptor, the second between OXT9 of the ligand and Asn 6841 in the pocket, the third between O4’ of the ligand and Tyr 6930 in the pocket, the fourth between O2’ of the ligand and Asn 6899 in the pocket, and the fifth between N1 of the ligand and Cys 6913, with distances 2.85 Å, 2.76 Å, 3.21 Å, 2.87 Å and 3.22 Å, respectively, and with energy scores of −6.6 Kcal/mol, −2.3 Kcal/mol, −1.3 Kcal/mol, Ȓ0.7 Kcal/mol and −3.9 Kcal/mol, respectively. An -ionic bond between N1 of the ligand and Asp 6928 in the protein pocket with distance 2.69 Å and energy score of −6.9 Kcal/mol.

**Table 3:** In silico prediction of ADME/Tox profiles of the studied compounds.

Compound	Log Po/w (WLOGP)	Solubility Class	BBB Permanent	CYP3A4 Substrate	PAINS	hERG I Inhibitor
Moloka′Iamine (**1**)	2.44	Soluble	Yes	No	0 alert	No
Mololipids (**2**)	14.13	Insoluble	No	Yes	0 alert	No
Fistularin-3 (**3**)	2.97	Moderately soluble	No	No	0 alert	No
11-ketofistularin-3 (**4**)	3.18	Poorly soluble	No	No	0 alert	No
Psammaplysin D (**5**)	7.84	Insoluble	No	Yes	0 alert	No
Psammaplysene D (**6**)	7.00	poorly soluble	No	Yes	0 alert	No
Psammaplysene F (**7**)	6.38	Poorly soluble	No	Yes	0 alert	No
Psammaplysene G (**8**)	6.38	Poorly soluble	No	Yes	0 alert	No
Psammaplysene H (**9**)	6.66	Poorly soluble	No	Yes	0 alert	No
Psammaplysene I (**10**)	6.32	Poorly soluble	No	Yes	0 alert	No
Anomoian C (**11**)	5.95	Poorly soluble	No	Yes	0 alert	No
Anomoian D (**12**)	5.6	Poorly soluble	No	Yes	0 alert	No
Anomoian E (**13**)	5.95	Poorly soluble	No	Yes	0 alert	No
Anomoian F (**14**)	6.57	Poorly soluble	No	Yes	0 alert	No

## Data Availability

Not applicable.

## Supplementary Materials

The following are available online. Table S1: Final docking validation, Figure S1: 3D interactions between compounds **1**–**14** and the native ligand with MPro (PDB ID: 6lu7), Figure S2: 3D interactions of the 14 library compounds in addition to the native ligand with spike glycoprotein (PDB ID: 6VYB), Figure S3: 3D interactions of the 14 library compounds plus the native ligand with nucleocapsid phosphoprotein (PDB ID: 6VYO), Figure S4: 3D interactions between the 14 library compounds with membrane glycoprotein (PDB ID: 6M17), Figure S5: 3D interactions of the 14 test compounds and the native ligand with the non-structural protein, nsp10 (PDB ID: 6W4H).

## Sample Availability

Samples of the compounds are not available from the authors.
