# Supplementary material for: Marine Brominated Tyrosine Alkaloids as Promising Inhibitors of SARS-CoV-2
Source: Molecules. 2021 Oct 13;26(20):6171. doi: 10.3390/molecules26206171 (PMC8537272; doi:10.3390/molecules26206171)
Supplement: Supplementary file 1 [file molecules-26-06171-s001.zip › molecules-1378511-supplementary.pdf]

Supporting information

Marine Brominated Tyrosine Alkaloids as Promising Inhibitors of SARS-CoV-2

Amr El-Demerdash [1,2,#,*], Afnan Hassan [3,4,#], Tarek Mohamed Abd El-Aziz [5,6], James D. Stockand [6,*] and Reem K. Arafa [3,4,*]

[1] Metabolic Biology & Biological Chemistry Department, The John Innes Center, Norwich Research Park, Norwich, NR4 7UH, Norwich, UK; eldemerdash555@gmail.com

[2] Organic Chemistry Division, Chemistry Department, Faculty of Science, Mansoura University, Mansoura 35516, Egypt

[3] Drug Design and Discovery Lab, Helmy Institute for Medical Sciences, Zewail City of Science and Technology, 12578, Egypt; afhassan@zewailcity.edu.eg; rkhidr@zewailcity.edu.eg

[4] Biomedical Sciences Program, University of Science and Technology, Zewail City of Science and Technology, 12578, Egypt

[5] Zoology Department, Faculty of Science, Minia University, El-Minia 61519, Egypt; mohamedt1@uthscsa.edu

[6] Department of Cellular and Integrative Physiology, University of Texas Health Science Center at San Antonio, San Antonio, TX 78229, USA; stockand@uthscsa.edu

[#] Authors with equal contribution

* Correspondence: eldemerdash555@gmail.com (A.E.-D.); Stockand@uthscsa.edu (J.D.S.); rkhidr@zewailcity.edu.eg (R.K.A)

Table of Contents

Table S1: Final docking validation 3

Three Dimensional Structures 5

Figure S1: 3D interactions between compounds **1-14** and the native ligand with M^{Pro} (PDB ID: 6lu7). 5

Figure S2. 3D interactions of the 14 library compounds in addition to the native ligand with spike glycoprotein (PDB ID: 6VYB). .. 8

Figure S3. 3D interactions of the 14 library compounds plus the native ligand with nucleocapsid phosphoprotein (PDB ID: 6VYO). 11

Figure S4. 3D interactions between the 14 library compounds with membrane glycoprotein (PDB ID: 6M17). 14

Figure S5. 3D interactions of the 14 test compounds and the native ligand with the non-structural protein, nsp10 (PDB ID: 6W4H). 17

Table S1: Final docking validation

PDB ID	Co-crystallized pose	Re-docked pose	Overlay Complex/RMSD (kcal/mol)
6LU7	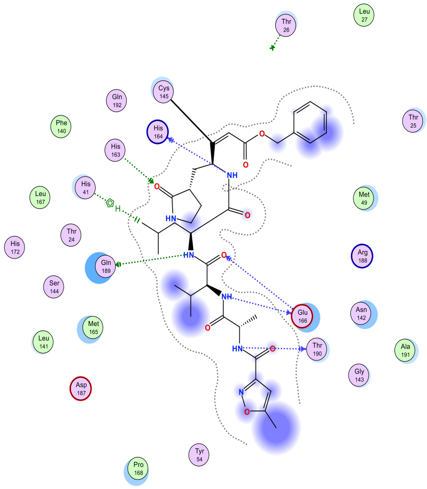	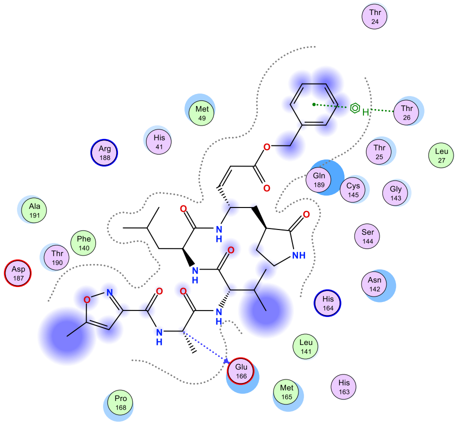	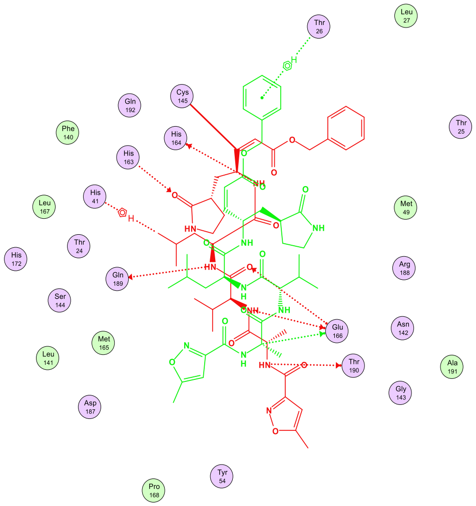 2.009
6VYB	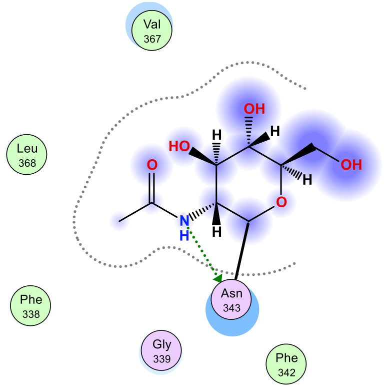	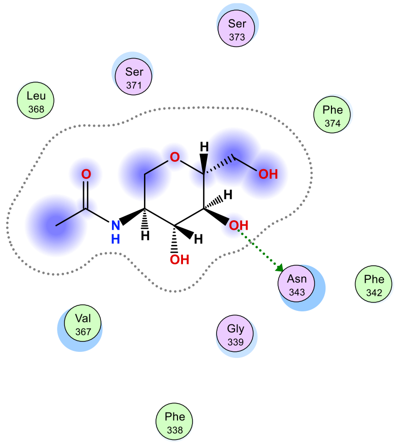	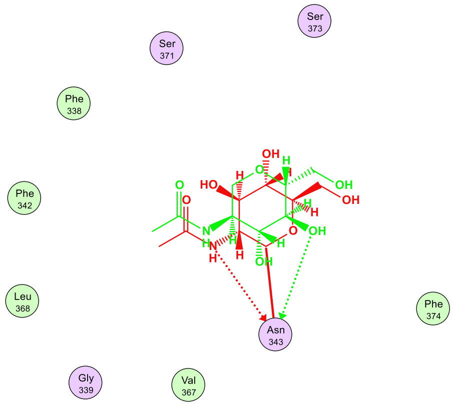 0.8360

6VYO

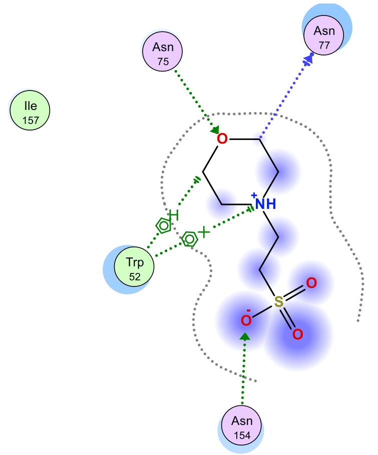

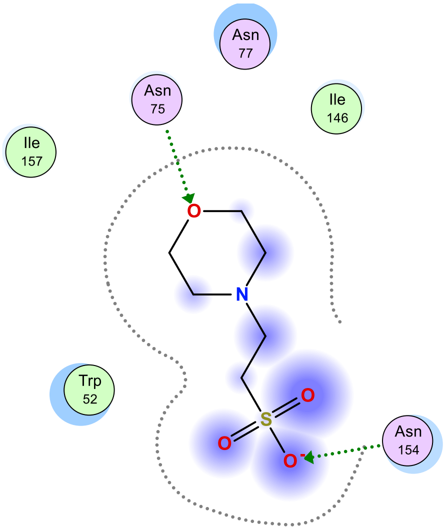

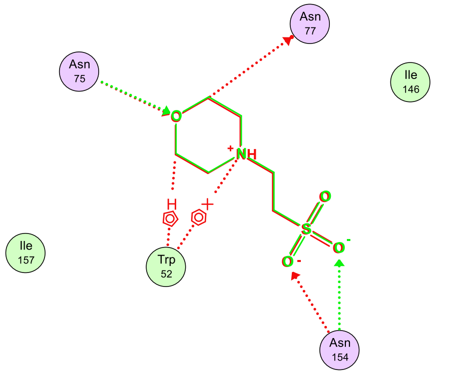

0.856

6W4 H

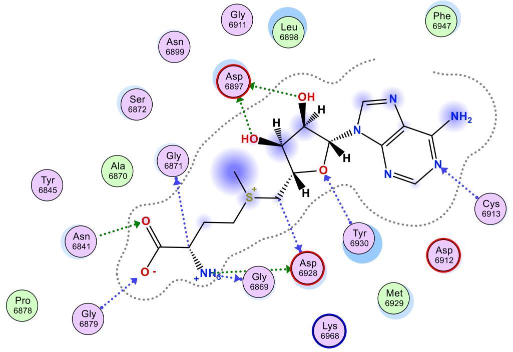

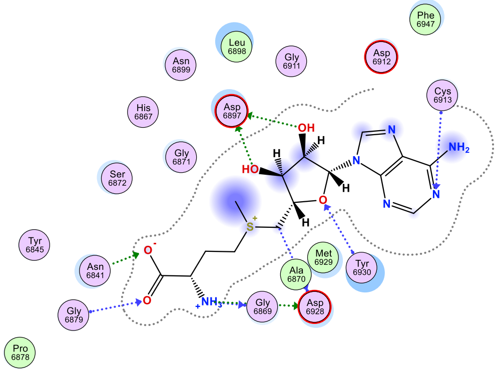

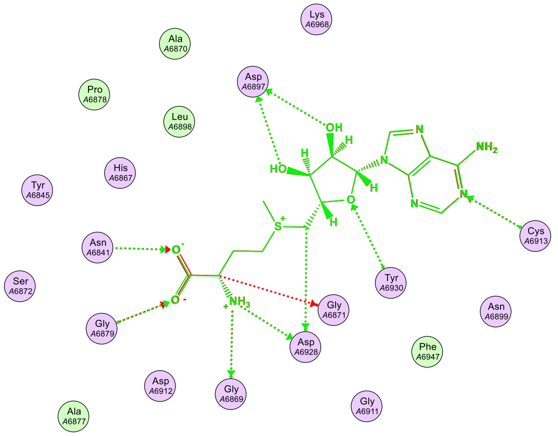

0.4512

Three Dimensional Structures

Figure S1: 3D interactions between compounds **1-14** and the native ligand with M^{Pro} (PDB ID: 6lu7).

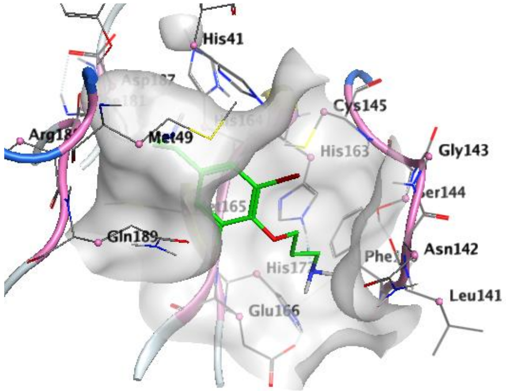

(1)

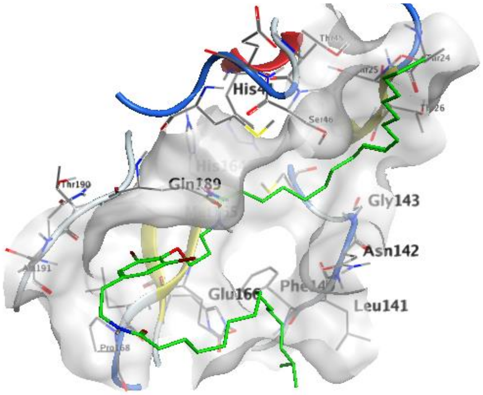

(2)

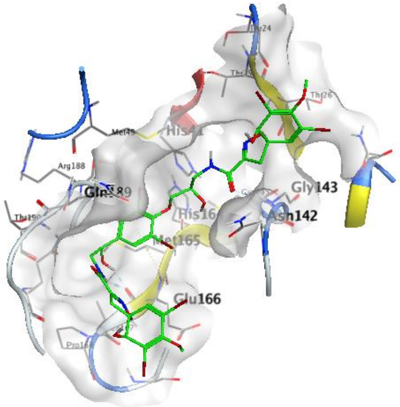

(3)

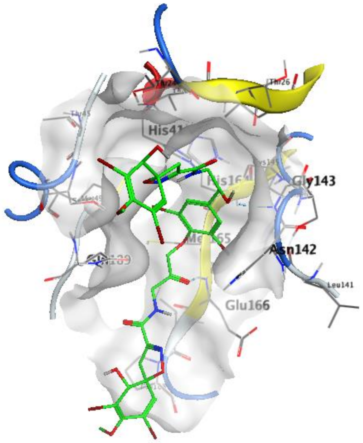

(4)

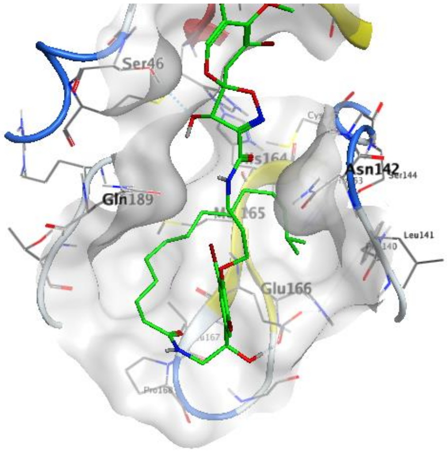

(5)

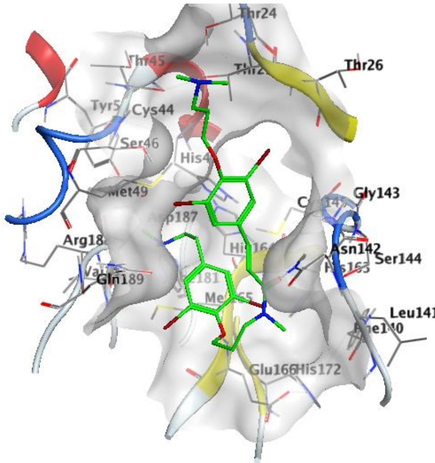

(6)

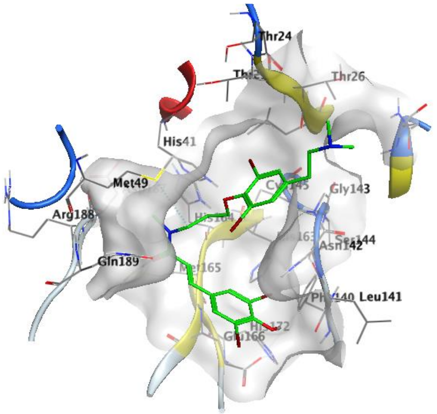
Thr24
Thr26
His41
Met49
Arg188
Gln189
Met165
Gly143
Ser144
Asn142
Leu141
Glu166

(7)

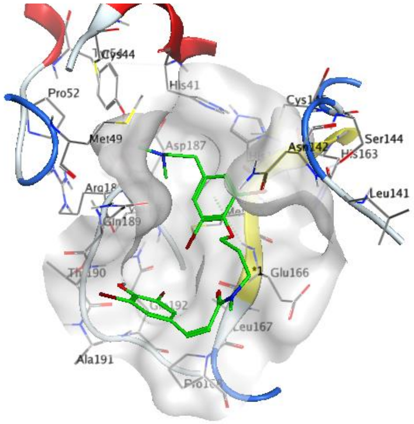
Cys44
Pro52
His41
Met49
Asp187
Asn142
Ser144
His163
Leu141
Gln189
Thr190
Glu166
Leu167
Ala191

(8)

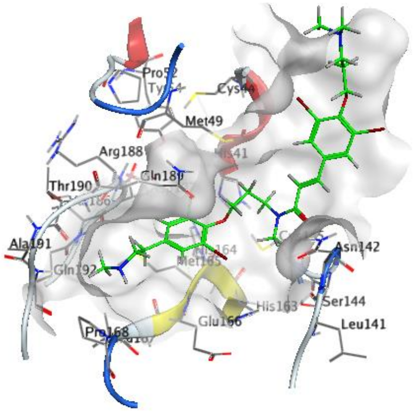
Pro52
Cys44
Met49
Arg188
Gln189
Thr190
Ala191
Gln192
Met165
Asn142
His163
Ser144
Leu141
Pro168
Glu166

(9)

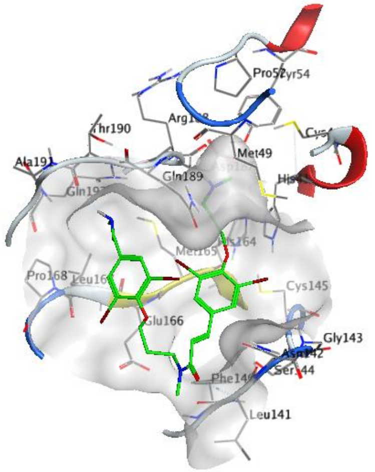
Pro52
Thr190
Ala191
Met49
Gln189
Pro168
Cys145
Glu166
Gly143
Asn142
Ser144
Phe140
Leu141

(10)

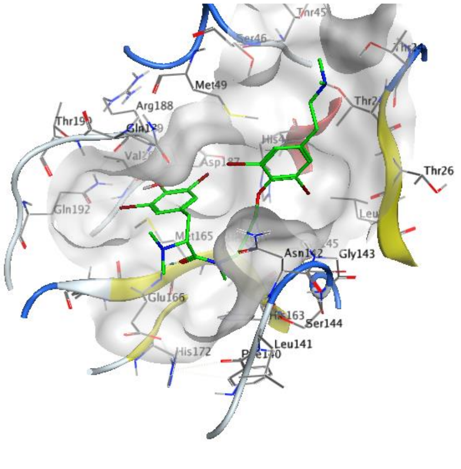
Thr45
Ser46
Met49
Arg188
Thr190
Gln192
Asp187
Thr26
Met165
Asn142
Gly143
Glu166
His163
Ser144
His172
Leu141

(11)

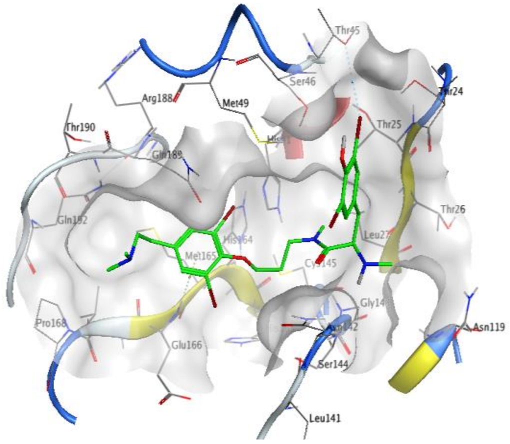
Thr45
Ser46
Arg188
Met49
Thr190
Gln189
Thr24
Thr25
Gln192
Thr26
Met165
His164
Cys145
Pro168
Glu166
Ser144
Asn119
Leu141

(12)

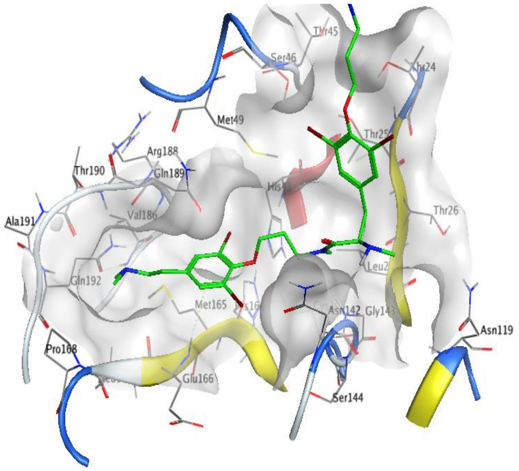
Tyr45
Ser46
Met49
Arg188
Thr190
Gln189
Thr24
Thr25
Thr26
Ala191
Val186
Gln192
Met165
Asn142
Gly143
Asn119
Pro168
Glu166
Ser144

(13)

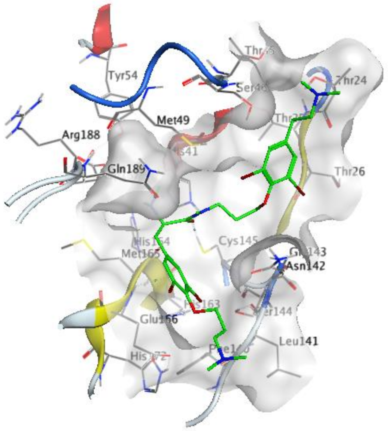
Tyr54
Met49
Arg188
Gln189
Thr24
Thr26
Met165
Cys145
Asn142
Glu166
Leu141

(14)

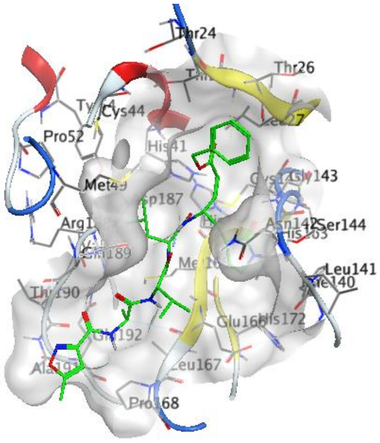
Thr24
Thr26
Cys44
Pro52
His41
Met49
Ser144
Leu141
Thr190
His172
Leu167
Pro168

(Native)

Figure S2. 3D interactions of the 14 library compounds in addition to the native ligand with spike glycoprotein (PDB ID: 6VYB).

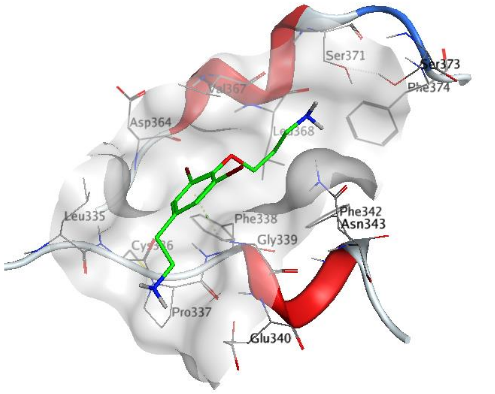

(1)

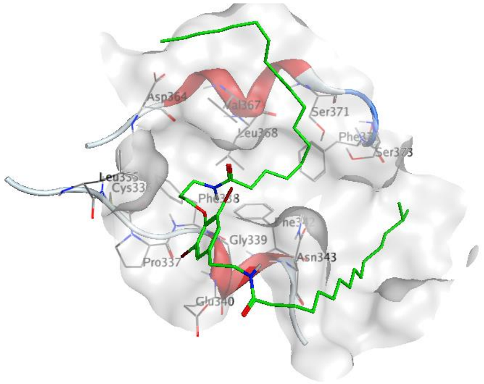

(2)

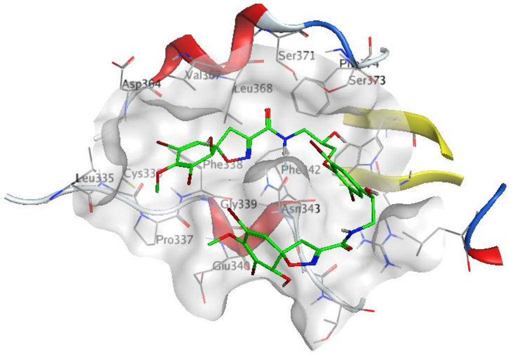

(3)

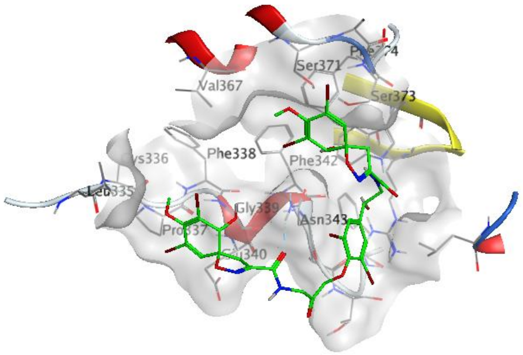

(4)

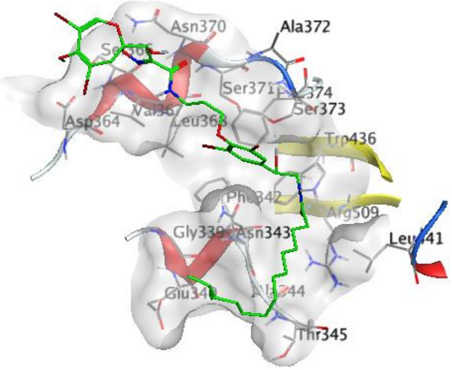

(5)

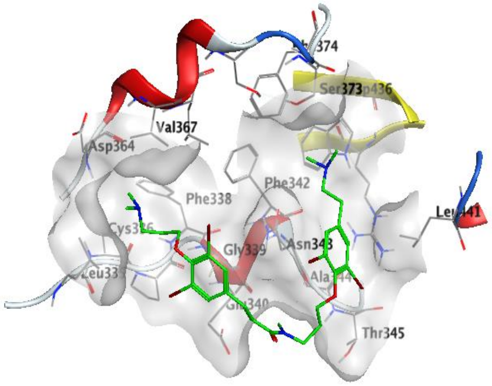

(6)

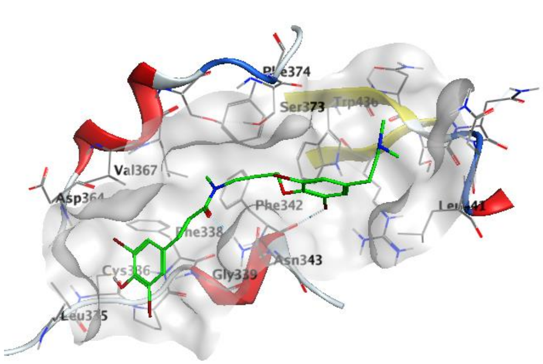
Phe374
Ser373
Trp436
Val367
Asp364
Phe342
Phe338
Leu441
Asn343
Gly339
Cys336
Leu335

(7)

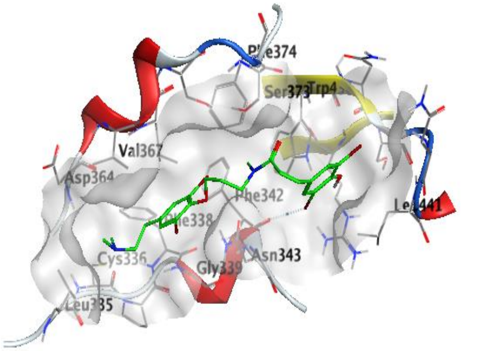
Phe374
Val367
Asp364
Phe342
Phe338
Asn343
Gly339
Cys336
Leu441

(8)

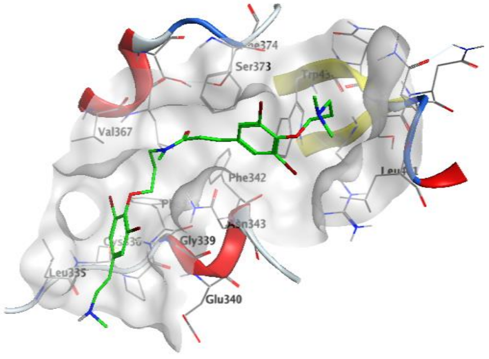
Ser373
Val367
Phe342
Gly339
Leu335
Glu340

(9)

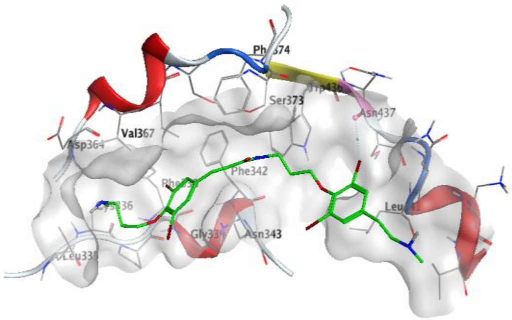
Ser373
Asn437
Asp364
Phe342
Asn343

(10)

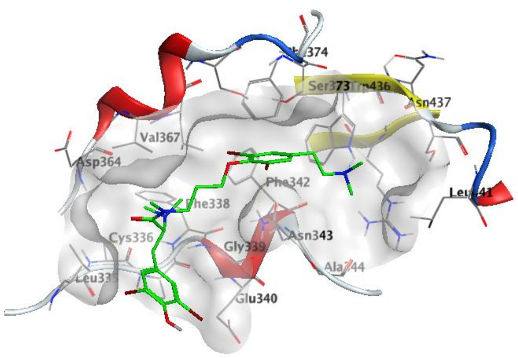
Trp436
Asn437
Val367
Asp364
Phe342
Phe338
Asn343
Gly339
Cys336
Glu340

(11)

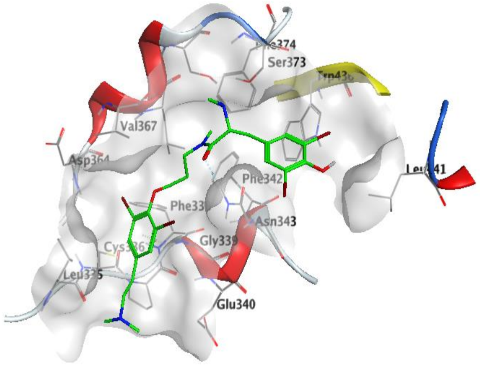
Ser373
Val367
Phe342
Asn343
Gly339
Glu340

(12)

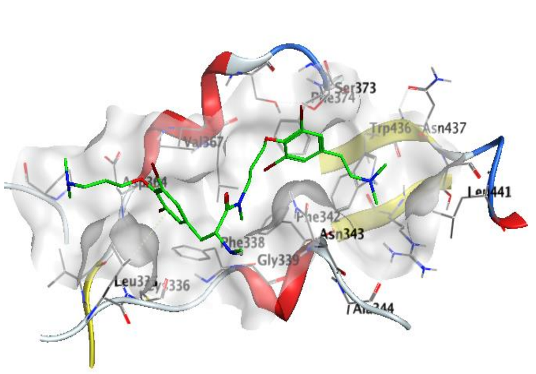
Ser373
Phe374
Val367
Trp436
Asn437
Leu441
Phe342
Asn343
Phe338
Gly339

(13)

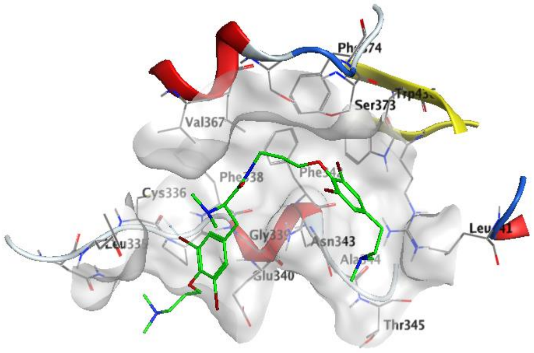
Val367
Ser373
Cys336
Phe338
Asn343
Glu340
Thr345

(14)

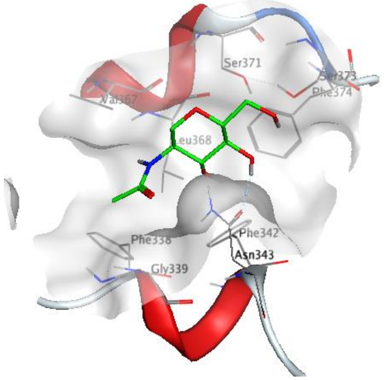
Ser371
Ser373
Phe374
Phe338
Gly339
Phe342
Asn343

(Native)

Figure S3. 3D interactions of the 14 library compounds plus the native ligand with nucleocapsid phosphoprotein (PDB ID: 6VYO).

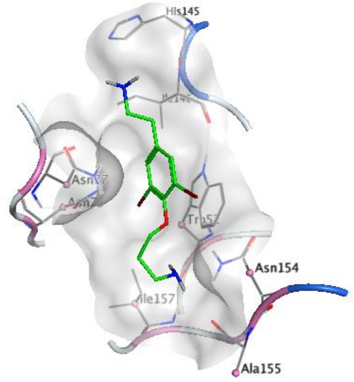

(1)

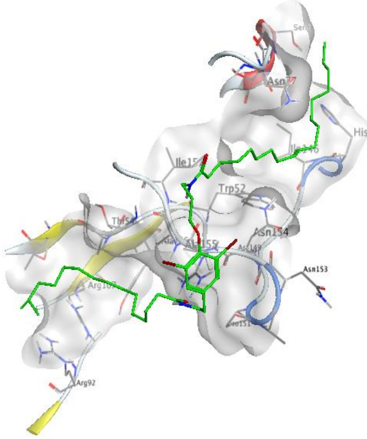

(2)

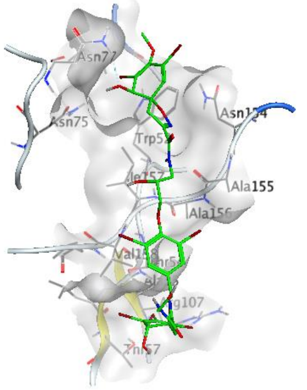

(3)

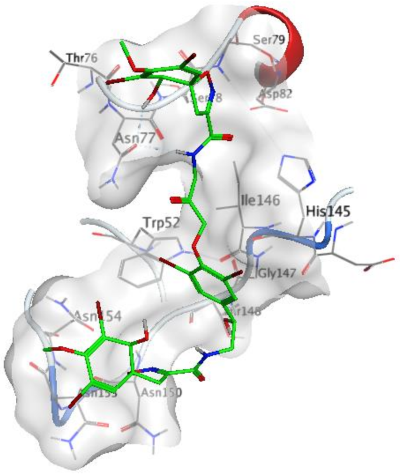

(4)

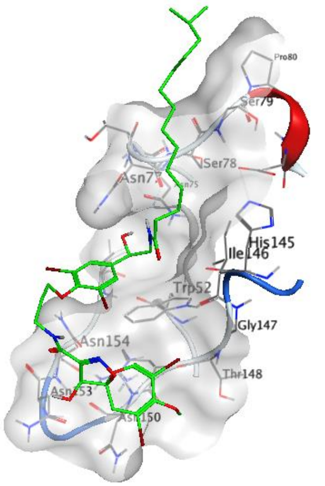

(5)

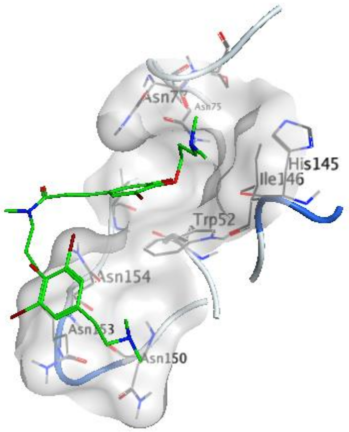

(6)

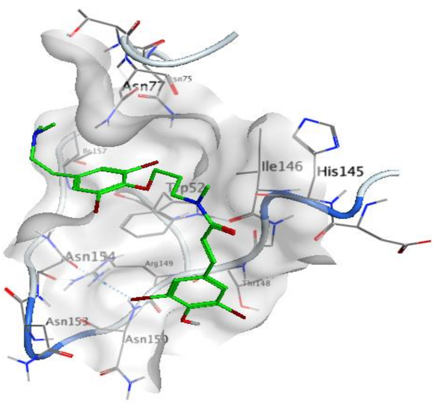
Asn77
Ile146
His145
Trp52
Asn154
Asn153
Asn150

(7)

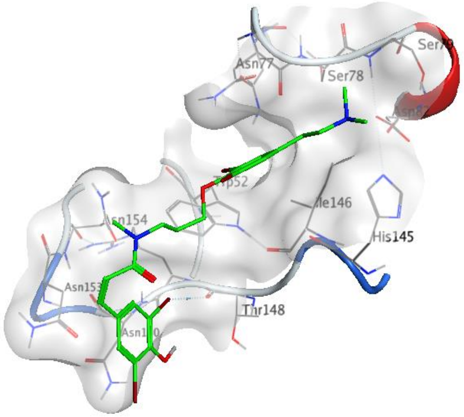
Asn77
Ser78
Ser79
Trp52
Ile146
His145
Asn154
Asn153
Thr148

(8)

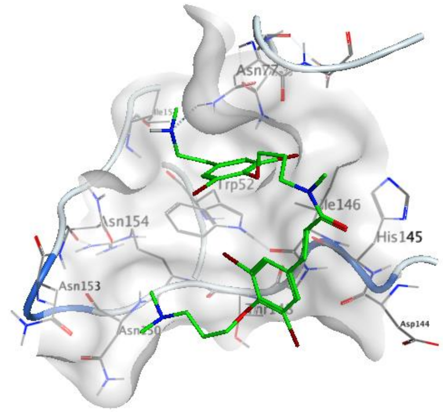
Asn77
Trp52
Ile146
His145
Asn154
Asn153
Asp144

(9)

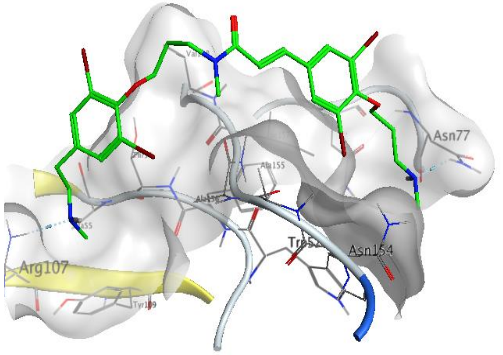
Asn77
Arg107
Trp52
Asn154

(10)

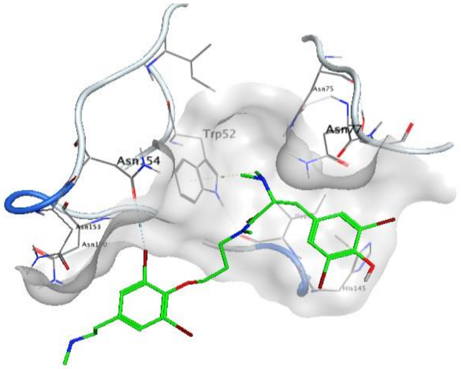
Trp52
Asn77
Asn154

(11)

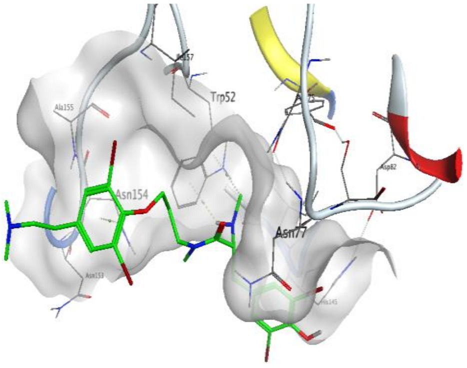
Trp52
Asn154
Asn77

(12)

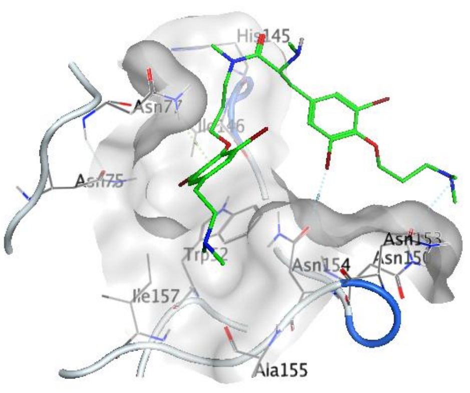
His145
Asn75
Ile157
Asn154
Asn153
Ala155

(13)

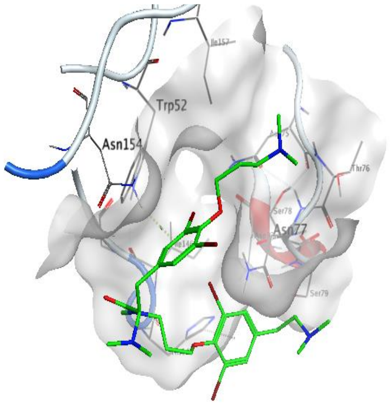
Trp52
Asn154
Asn77

(14)

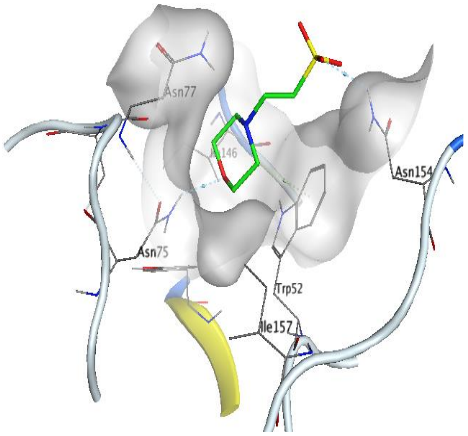
Asn77
Asn154
Asn75
Trp52
Ile157

(Native)

Figure S4. 3D interactions between the 14 library compounds with membrane glycoprotein (PDB ID: 6M17).

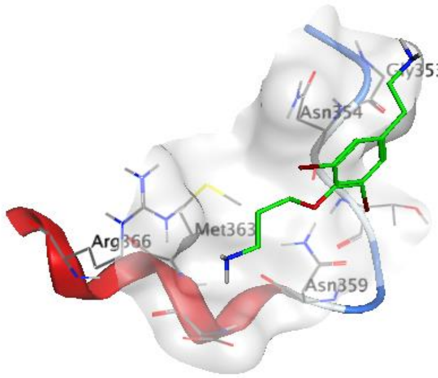

(1)

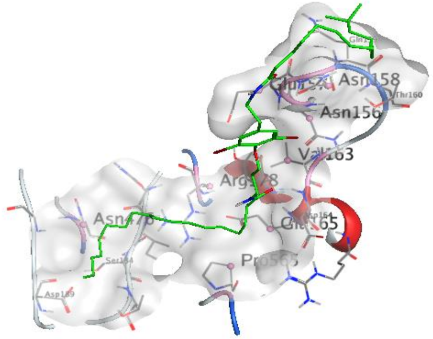

(2)

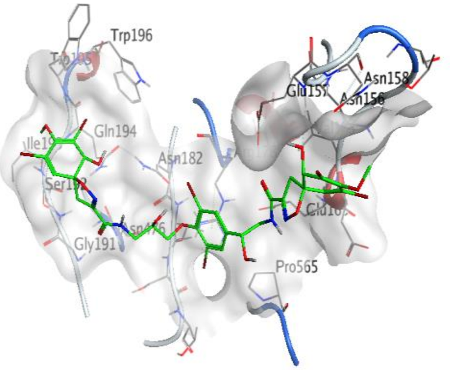

(3)

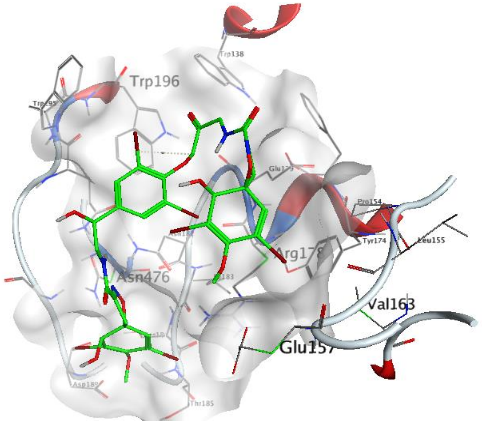

(4)

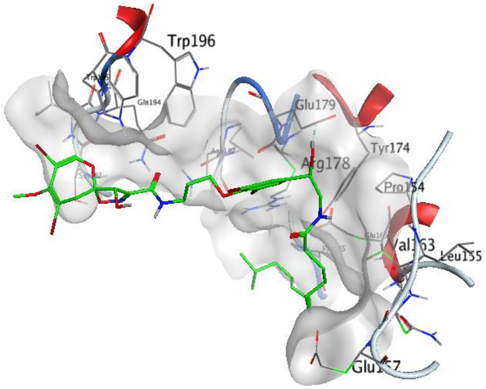

(5)

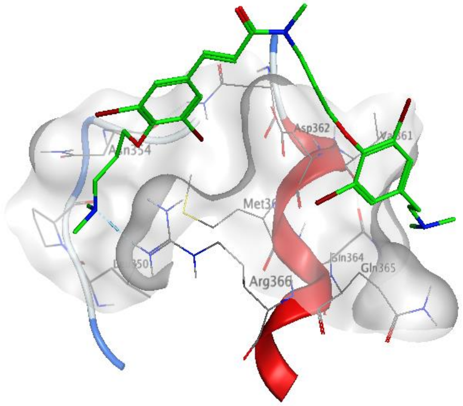

(6)

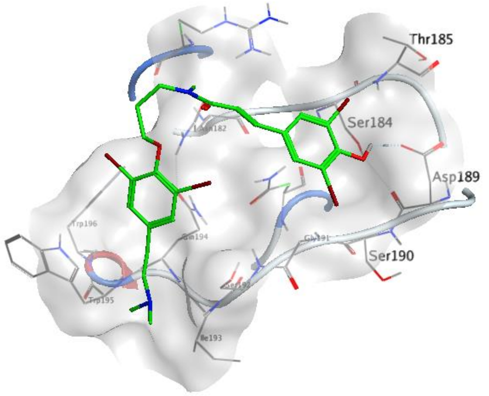
Thr185
Ser184
Asp189
Ser190

(7)

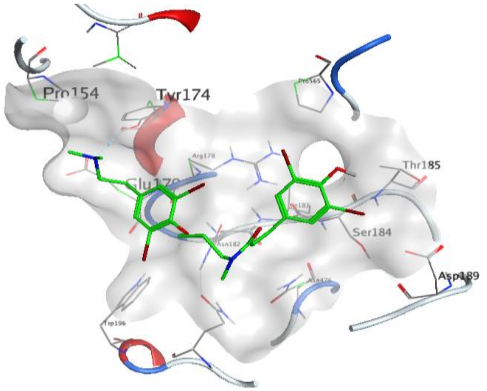
Pro154
Tyr174
Thr185
Ser184
Asp189

(8)

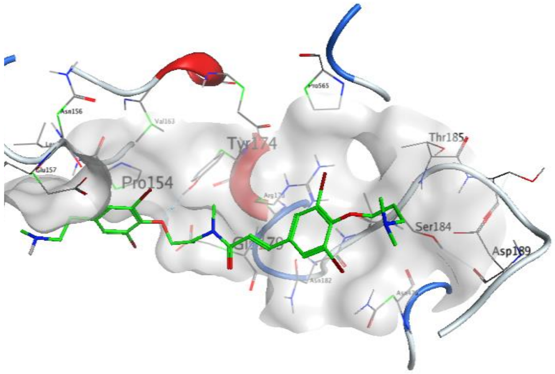
Tyr174
Pro154
Thr185
Ser184
Asp189

(9)

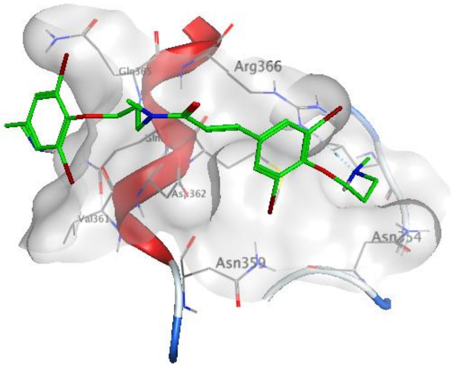
Arg366
Asn354

(10)

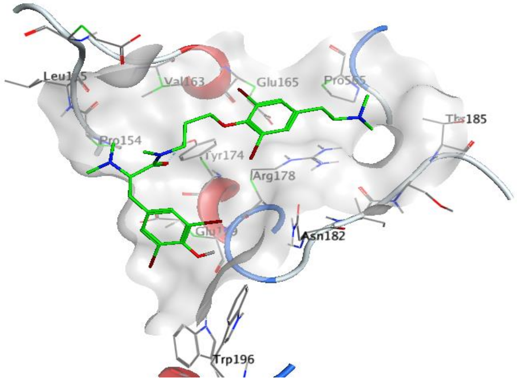
Glu165
Pro154
Tyr174
Arg178
Thr185
Asn182
Trp196

(11)

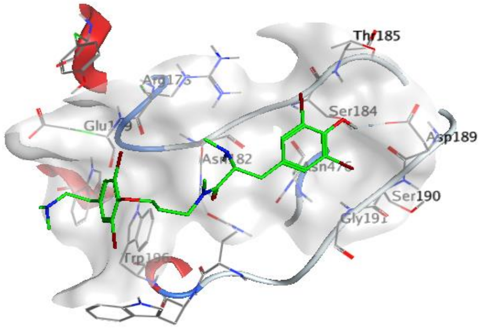
Thr185
Ser184
Asp189
Ser190
Gly191

(12)

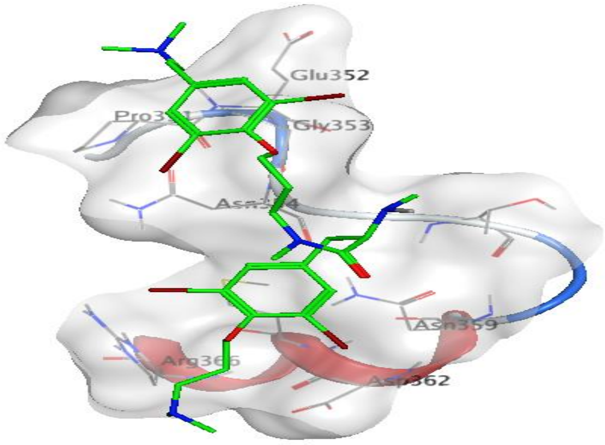

Glu352
Gly353
Asn359
Arg365
Asp362

(13)

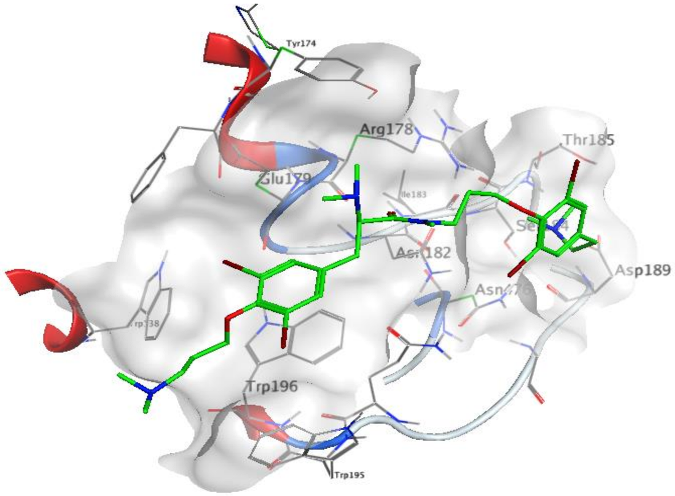

Tyr174
Arg178
Thr185
Glu179
Asp189
Asn182
Trp196
Trp195

(14)

Figure S5. 3D interactions of the 14 test compounds and the native ligand with the non-structural protein, nsp10 (PDB ID: 6W4H).

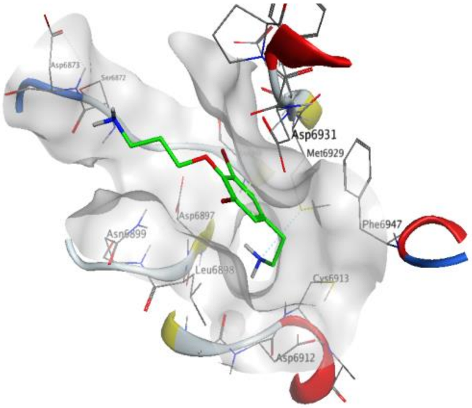

(1)

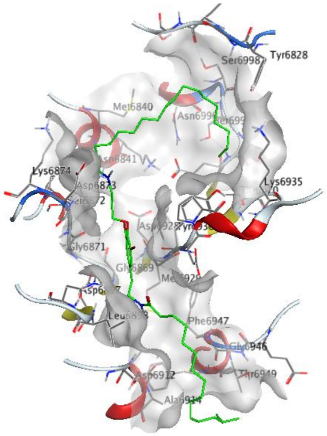

(2)

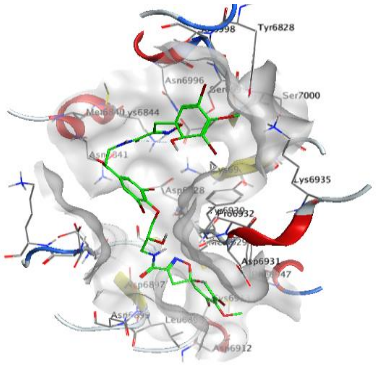

(3)

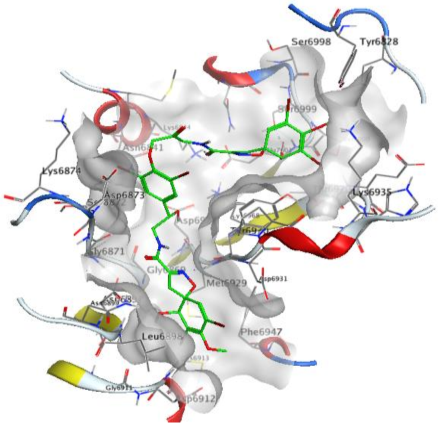

(4)

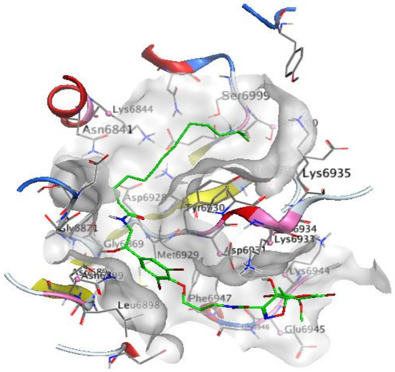

(5)

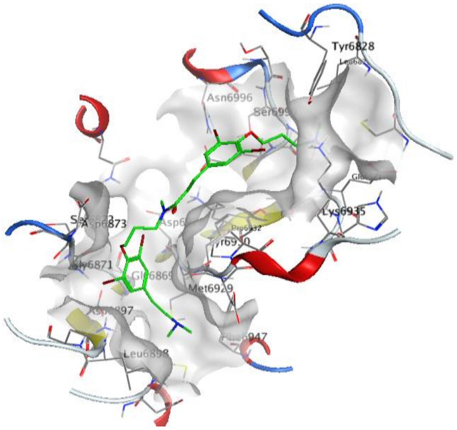

(6)

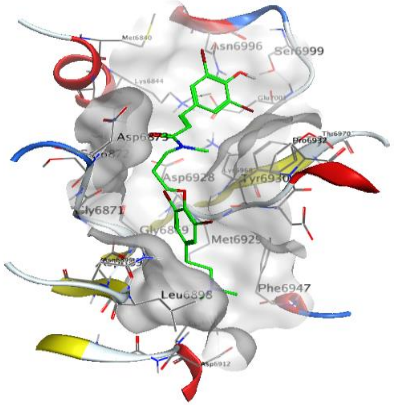
Asn6996
Ser6999
Asp6873
Asp6928
Tyr6930
Gly6871
Met6929
Leu6898
Phe6947

(7)

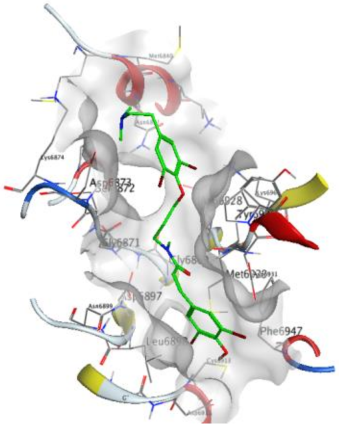
Phe6947

(8)

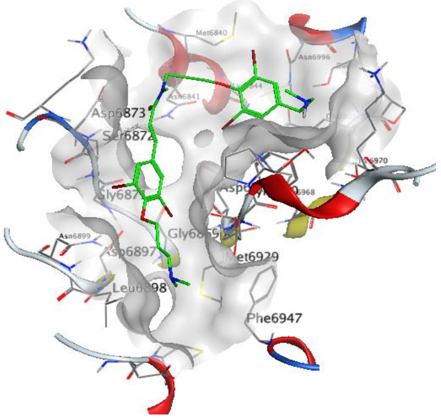
Asp6873
Asp6897
Leu6898
Met6929
Phe6947

(9)

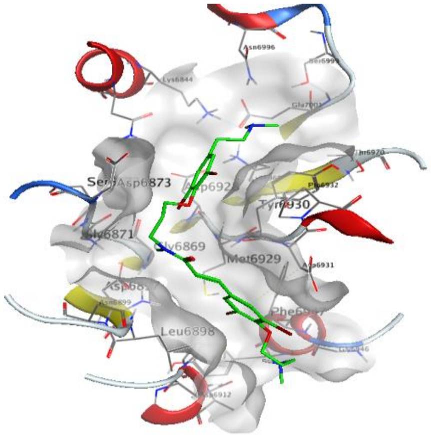
Asp6873
Gly6871
Gly6869
Tyr6930
Met6929
Leu6898

(10)

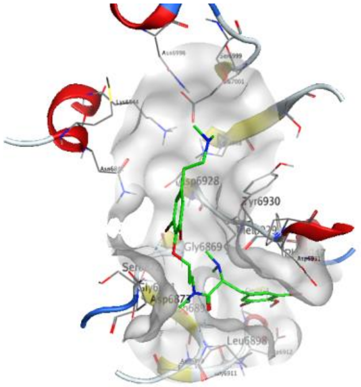
Asp6928
Tyr6930
Gly6869

(11)

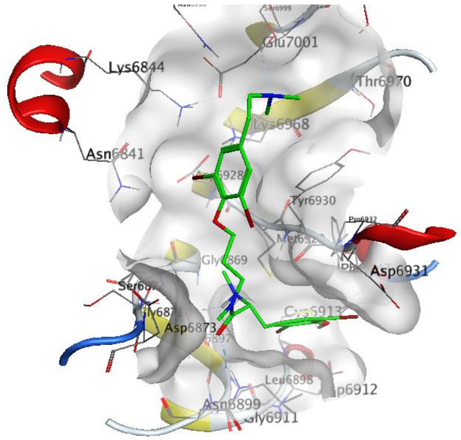
Glu7001
Lys6844
Thr6970
Lys6968
Asn6841
Tyr6930
Asp6931
Gly6869
Asp6873
Leu6898
Asn6899
Gly6911

(12)

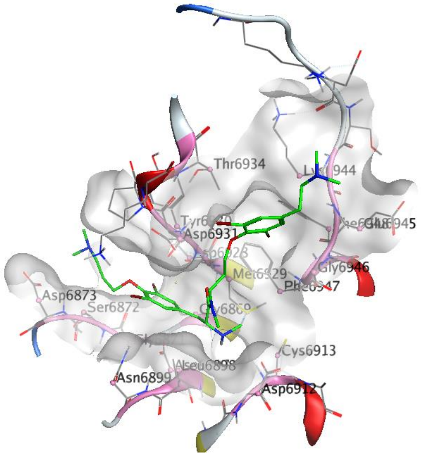
Thr6934
Asp6931
Asp6873
Ser6872
Cys6913
Asn6899
Asp6912

(13)

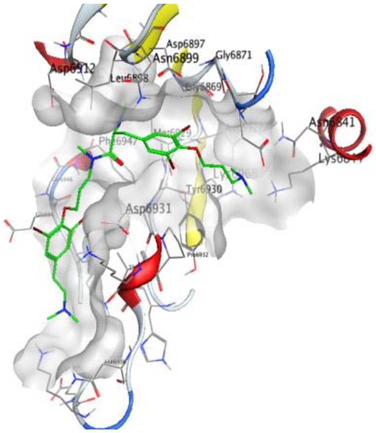
Asp6897
Asn6899
Gly6871
Asp6931

(14)

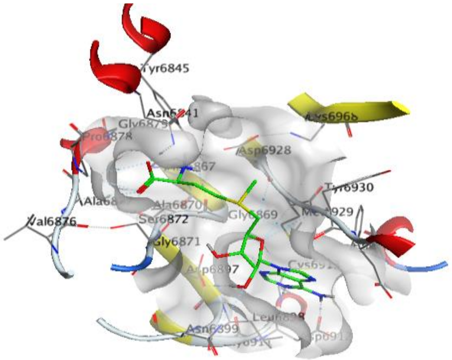
Tyr6845
Asp6928
Tyr6930
Val6876
Ser6872
Gly6871
Asn6899

(Native)
